# DMRT1 regulates human germline commitment

**DOI:** 10.1038/s41556-023-01224-7

**Published:** 2023-09-14

**Authors:** Naoko Irie, Sun-Min Lee, Valentina Lorenzi, Haiqi Xu, Jinfeng Chen, Masato Inoue, Toshihiro Kobayashi, Carmen Sancho-Serra, Elena Drousioti, Sabine Dietmann, Roser Vento-Tormo, Chun-Xiao Song, M. Azim Surani

**Affiliations:** 1 Wellcome Trust/Cancer Research UK Gurdon Institute, Henry Wellcome Building of Cancer and Developmental Biology, Cambridge, UK; 2 Metabolic Systems Laboratory, Live Imaging Center, Central Institute for Experimental Animals, Kanagawa, Japan; 3 Department of Physics, Konkuk University, Seoul, Republic of Korea; 4 Wellcome Sanger Institute, Cambridge, UK; 5 European Molecular Biology Laboratory, European Bioinformatics Institute, Cambridge, UK; 6 Ludwig Institute for Cancer Research and Target Discovery Institute, Nuffield Department of Medicine, University of Oxford, Oxford, UK; 7 Division of Mammalian Embryology, Center for Stem Cell Biology and Regenerative Medicine, The Institute of Medical Science, The University of Tokyo, Tokyo, Japan; 8 Center for Genetic Analysis of Behavior, National Institute for Physiological Sciences, Aichi, Japan; 9 Department of Developmental Biology and Institute for Informatics, Washington University School of Medicine, St. Louis, MO USA; 10 Physiology, Development and Neuroscience Department, University of Cambridge, Cambridge, UK

**Keywords:** Embryonic germ cells, Germline development, Epigenetics, Embryonic stem cells

## Abstract

Germline commitment following primordial germ cell (PGC) specification during early human development establishes an epigenetic programme and competence for gametogenesis. Here we follow the progression of nascent PGC-like cells derived from human embryonic stem cells in vitro. We show that switching from BMP signalling for PGC specification to Activin A and retinoic acid resulted in DMRT1 and CDH5 expression, the indicators of migratory PGCs in vivo. Moreover, the induction of DMRT1 and SOX17 in PGC-like cells promoted epigenetic resetting with striking global enrichment of 5-hydroxymethylcytosine and locus-specific loss of 5-methylcytosine at DMRT1 binding sites and the expression of DAZL representing DNA methylation-sensitive genes, a hallmark of the germline commitment programme. We provide insight into the unique role of DMRT1 in germline development for advances in human germ cell biology and in vitro gametogenesis.

## Abstract (web summary)

Irie, Lee et al. report a role for DMRT1 in human germline development and show that induction of DMRT1 in primordial germ cell-like cells triggers germline commitment, but suppresses pluripotency genes, thus promoting the onset of gametogenesis.

## Main

Germ cells generate a totipotent zygote state at fertilization and transmit genetic and epigenetic information for development to term^1^. In humans, primordial germ cells (PGCs), the precursors of eggs and sperm, appear on approximately week 2 in gastrulating embryos^2–4^. The subsequent migration of PGCs into gonads over approximately weeks 5 to 6 is accompanied by critical epigenetic resetting^5–10^. A prolonged development and dormancy follow before functionally mature sperm and eggs form at puberty^2–4^.

Technical and ethical reasons hamper research on early human PGCs, but in vitro models using human pluripotent stem (PS) cells, embryonic stem (ES) cells or induced PS cells have allowed advances in human germline biology, including the mechanism of PGC specification^4,11–15^. PS cells gain competence for germline fate following culture with GSK3 inhibitor and Activin A (ActA) called precursor of mesendoderm (preME)/incipient mesoderm-like cells (iMeLCs)^11,13^, or with four inhibitors for GSK3, MEK, p38, JNK with FGF2, TGFβ and LIF (henceforth called 4i ES cells)^14^, that differentiate into PGC-like cells (PGCLCs) in response to BMP2/BMP4. PGCLCs can also be induced directly in response to the ectopic expression of SOX17 and PRDM1, the essential regulators of human PGC fate^13,14^. The molecular programme of PGC specification is conserved in mammals with bilaminar disc embryos^13,16–22^.

Development beyond the nascent stage has been explored with PGCs from aborted foetuses^5–10^, but infrequently from week 2 to week 6, because of their scarcity. The nascent PGCs in vivo originating at gastrulation, proliferate and migrate through the yolk sac endoderm, hindgut and dorsal mesentery before they reach developing gonads from approximately week 5 onwards^2–4^. Critical epigenetic resetting accompanies PGC migration, including a transient enrichment for DNA 5-hydroxymethylcytosine (5hmC) and progressive global loss of 5-methylcytosine (5mC) that diminishes to ~5% by week 9 (refs. ^6–9^). The expression of *DAZL*, *PIWIL1* and *PIWIL2* in PGCs as they enter gonads marks lineage commitment, while the expression of nascent PGC genes, *SOX17*, *PRDM1*, *NANOS3*, *POU5F1* and *NANOG* continues^6,23,24^. With germline commitment, there is suppression of pluripotency genes after proliferation when female PGCs commence meiosis followed by oogenesis at approximately weeks 11 to 14, and male PGCs undergo mitotic arrest at approximately week 9 (ref. ^24^). Failure of the commitment may lead to germ cell tumourigenesis^25,26^.

Nascent PGCLCs induced from PS cells exhibit the potential to develop further, albeit at a low frequency, when co-cultured with hindgut organoids^27^ or mouse gonadal somatic cells^28,29^. However, the underlying mechanisms of development, epigenetic resetting, global DNA demethylation and migratory/gonadal PGC gene expression, including DAZL, remain unclear.

In this Article, we report the development of PGCLCs beyond the nascent stage in defined conditions and reveal the hitherto unknown role of DMRT1 in the transition from nascent PGCs towards germline commitment. DMRT1, with a DM domain and zinc finger-like DNA binding motif, is evolutionarily conserved and is best known for its role in sex determination and spermatogenesis^30–33^. Our study provides mechanistic insights into the role of DMRT1 in the stepwise developmental progression of human germ cell lineage.

## Results

### Sustained signalling for PGCLCs restricts progression

**Fig. 1:**
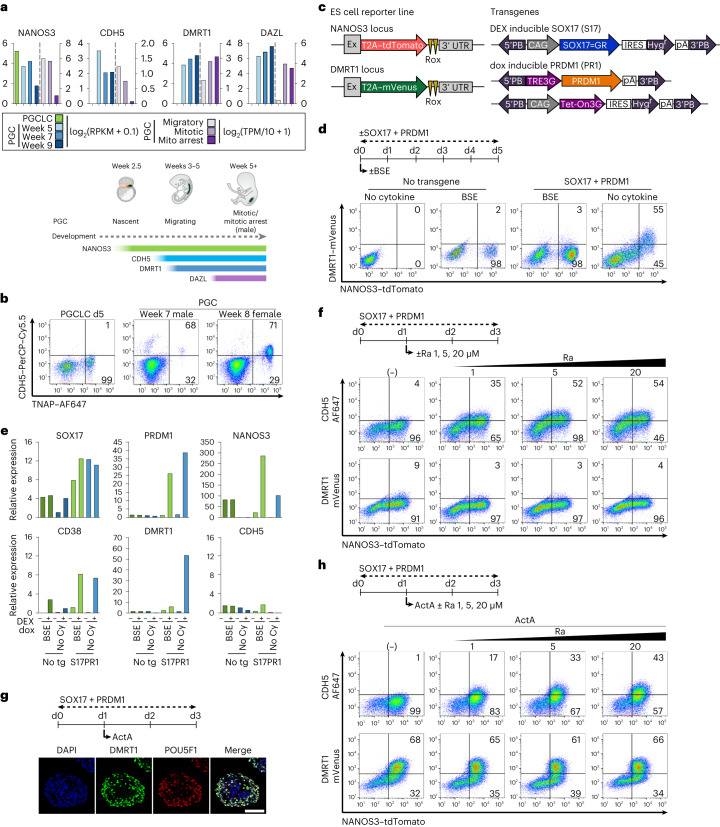
Sustained signalling for PGCLC specification restricts subsequent development. **a**, RNA-seq data^6,14,24^ of in vitro induced day 4 PGCLCs and in vivo PGCs. Top: bar plot represents mean (PGC weeks 5/7/9, *n* = 2 biological samples; migratory, 37 cells; mitotic, 332 cells; mitotic arrest, 309 cells). Bottom: stage-specific gene expression during human PGC development. Vertical dashed lines separate the two datasets based on their different vertical scales. **b**, Flow cytometry of CDH5 and TNAP for in vitro induced day (d)5 PGCLCs and in vivo PGCs from week 7 male and week 8 female. Values are percentage ratio of CDH5-positive and CDH5-negative population in TNAP-expressing cells. **c**, PiggyBac (PB) gene introduction for DEX-inducible SOX17=GR and dox-inducible PRDM1 in NANOS3–tdTomato/DMRT1–mVenus double reporter ES cells. **d**,**e**, Flow cytometry (**d**) and RT–qPCR (**e**) of PGCLC induction from parental NANOS3–tdTomato/DMRT1-mVenus reporter WIS2 ES cells (no transgene, no tg) and SOX17=GR/TRE–PRDM1 cell line (S17PR1) clone 1 treated with (SOX17 + PRDM1, +) or without (−) DEX and dox for 5 days with BSE (BMP2, SCF and EGF) or without cytokines (no cytokine, no Cy). Values in **d** are percentage ratio of DMRT1–mVenus-positive and DMRT1–mVenus-negative population in NANOS3–tdTomato-expressing cells. Values in **e** are normalized with GAPDH and relative changes against no tg/no Cy/DEXdox (−). A repeat experiment with independent clone with similar results shown in Extended Data Fig. 1f. **f**, Flow cytometry of NANOS3–tdTomato against DMRT1–mVenus and CDH5–AF647. Induction of SOX17 + PRDM1 for 3 days, with (1, 5, 20 µM) or without (−) Ra from day 2. Percentage ratio of CDH5 and DMRT1 in NANOS3–tdTomato-positive cells. **g**, Immunofluorescence for DMRT1 and POU5F1 co-staining on day 3 of SOX17 + PRDM1 induction with ActA 100 ng ml^−1^ from day 1. Scale bar, 100 μm. The experiment was repeated independently two times with similar results. **h**, Flow cytometry of NANOS3–tdTomato against DMRT1–mVenus and CDH5–AF647. Induction of SOX17 + PRDM1 for 3 days with ActA 100 ng ml^−1^ from day 1 with (Ra; 1, 5, 20 µM) or without (−) Ra from day 1. Percentage ratio of CDH5 and DMRT1 in NANOS3–tdTomato-positive cells. Source data

**Extended Data Fig. 1:**
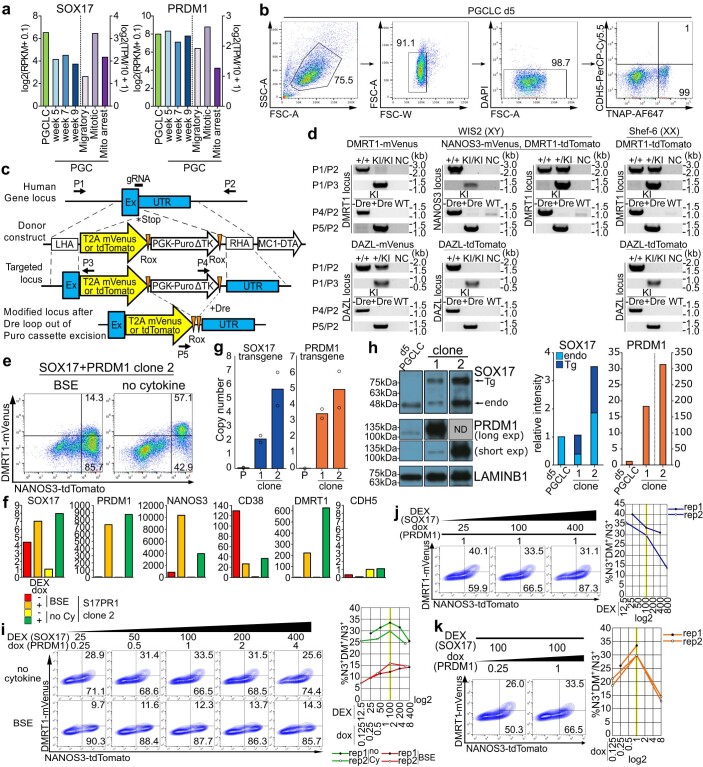
Sustained signaling for PGCLC specification restricts subsequent development. (**a**) RNA-sequencing data 6,14,24 of in vitro induced day 4 PGCLCs and in vivo PGCs for PGC genes. Bar plot represents mean values (PGC week5/7/9, n = 2 biological samples; migratory, 37 cells; mitotic, 332 cells; mitotic arrest, 309 cells). (**b**) Example of gating strategies for flowcytometry analysis with PGCLC d5 from Fig. 1b. Cell population is gated based on SSC-A/FSC-A, single cell population based on FSC-A/FSC-W and live cell population using DAPI. % gating populations/the parental populations. (**c**) Targeting strategy for reporter ES cells. Guide (g)RNA for CRISPR-Cas9 and genotyping primers (P1-P5) are indicated. (**d**) Genotyping PCR for reporter ES cells. +: wild type allele; KI: knock-in allele; NC: water control; WT: wildtype cells; −/+DRE: before (−) and after (+) puromycin resistant cassette excision. The experiment was repeated independently two times with similar results. (**e**) Flowcytometry and (**f**) RT-qPCR of PGCLC induction from NANOS3–tdTomato/DMRT1-mVenus double reporter male ES cells (WIS2) with inducible SOX17 = GR and TRE-PRDM1 cell line (clone 2) treated with DEX and dox (SOX17 + PRDM1) for five days in the presence or absence (no cytokine) of PGC specification cytokines, BMP2, SCF and EGF (BSE). Percentage for +/−DMRT1-mVenus in NANOS3-tdTomato positive cells are indicated. Values in (F) are normalized with the housekeeping gene GAPDH and relative changes against no tg/no Cy/DEXdox(-). A repeat experiment with independent clone with similar results is shown in Fig. 1e. (**g**) Quantitative PCR for copy number for genomic integration of the piggybac plasmids for SOX17 = GR and TRE-PRDM1 in clone 1 and 2. Biologically independent experiments, n = 2.(**h**) Western blot for expression of SOX17, PRDM1, LAMINB1 in the nuclear protein extract for the transgenic cell lines 1 and 2 in the presence of DEX and dox for 3 days, and PGCLCs for day 5 induced with cytokines, BMP2, SCF and EGF. The experiment was repeated independently two times with similar results. Dose response of DEX (μM) and dox (μg/ml) (**i**), DEX (**j**) and dox (**k**) for the expression of DMRT1 reporter in NANOS3+PGCLCs. Percentage for +/−DMRT1-mVenus in NANOS3-tdTomato positive cells are indicated. The graphs show the percentages from the two independent experiments. Source data

First, we compared the transcriptome of nascent PGCLCs (equivalent to weeks 2 to 3 PGCs in vivo) with the in vivo tissue non-specific alkaline phosphatase (TNAP)^+^KIT^+^ PGCs from week 5, week 7 and week 9 foetuses^6,14^. We classified PGC(LCs) as migratory, mitotic and mitotic arrest (gonocytes) as described previously (Methods)^24^. Nascent PGCLCs showed *SOX17*, *PRDM1* and *NANOS3* expression but without *CDH5* or *DMRT1*, which occurs in migratory PGCs of approximately week 4 (Fig. 1a and Extended Data Fig. 1a)^24^. We detected CDH5 in male and female gonadal PGCs (Fig. 1b and Extended Data Fig. 1b). Progressive upregulation of *DMRT1* in the mitotic and mitotic arrest PGCs was found, which was followed by *DAZL* expression, including subpopulation of migratory PGCs at week 5 (ref. ^27^), indicating a step-wise gene expression for germline commitment (Fig. 1a).

To monitor the progression of nascent PGCLCs, we established male and female ES cells with DMRT1 and NANOS3 dual fluorescent reporters (Fig. 1c and Extended Data Fig. 1c,d)^13,14^. These cells also contained dexamethasone (DEX)-inducible SOX17 and doxycycline (dox)-inducible PRDM1 factor for PGCLC induction without the cytokines (Fig. 1c)^13^, as well as anti-apoptotic BCL2L1.

The NANOS3–tdTomato-positive PGCLCs were induced in response to BMP2, SCF and EGF (BSE), or SOX17/PRDM1, with or without BSE after 5 days (Fig. 1d). Unexpectedly, SOX17/PRDM1 also robustly induced DMRT1 (55–57.1%) in the PGCLCs in the absence of BSE (Fig. 1d and Extended Data Fig. 1e–g). With the optimal concentrations of DEX and dox for SOX17 and PRDM1 induction, respectively, dose-dependent regulation of PRDM1 by dox affected the induction of DMRT1. However, with the lower concentration of DEX regulating SOX17, there was efficient induction of DMRT1, suggesting that appropriate levels of the two factors are essential for further development (Extended Data Fig. 1i–k). Quantitative reverse transcription polymerase chain reaction (RT–qPCR) confirmed *DMRT1* expression in response to SOX17 and PRDM1 without BSE in NANOS3^+^CD38^+^ PGCLCs, but *CDH5* expression was not detectable (Fig. 1e and Extended Data Fig. 1f), unlike in migratory PGCs (Fig. 1a,b). However, retinoic acid (Ra) induced CDH5 in a dose-dependent manner in PGCLCs generated in response to SOX17/PRDM1, but without the expression of DMRT1 reporter (Fig. 1f). Conversely, ActA induced DMRT1 in PGCLCs without CDH5 (Fig. 1g,h). Notably, the combination of ActA and Ra resulted in robust expression of both DMRT1 (−66%) and CDH5 (−43%) in PGCLCs (Fig. 1h), indicating that they act independently to induce expression of DMRT1 and CDH5 in nascent PGCLCs to promote a migratory state.

### Switching signalling promotes PGCLC progression

**Extended Data Fig. 2:**
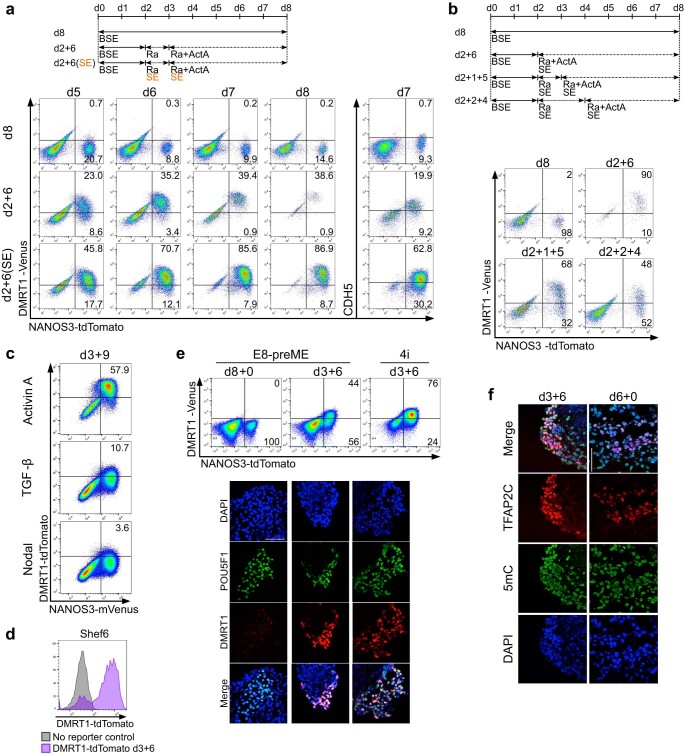
Switching signaling in nascent PGCLCs for further development. (**a** and **b**) Flowcytometry of NANOS3-tdTomato against DMRT1-mVenus. NANOS3-tdTomato/DMRT1-mVenus reporter 4i ES cells were induced PGCLCs with the cytokines indicated for 8 days. BSE: BMP2, SCF and EGF; Ra: retinoic acid, 20 μM; ActA: Activin A, 20 μg/ml; SE: SCF and EGF. Percentage populations of NANOS3 positive and DMRT1 positive or negative cells in live single cells are shown. (**c**) Flowcytometry of NANOS3-tdTomato against DMRT1-mVenus. 3-days induction of nascent PGCLCs with BSE followed by Ra 20 μM, SCF 100 μg/ml, EGF 50 μg/ml and, Activin A 100 μg/ml, TGFβ 100 μg/ml or Nodal 100 μg/ml. Percentage populations of NANOS3 and DMRT1 double positive cells in live cell population are shown. (**d**) Flowcytometry histogram of DMRT1-tdTomato. DMRT1-tdTomato female reporter line (Shef-6) was induced nascent PGCLCs with BSE for 3 days and followed by Ra 20 μM+Activin A 100 μg/ml+SCF 100 μg/ml+EGF 50 μg/ml for 6 days (d3 + 6). Parental Shef-6 ES cells without reporter was used as a negative control. (**e**) Flowcytometry and immunofluorescence for PGCLCs induced with BSE (d8 + 0) or BSE 3 days followed by RASE (d3 + 6) from conventional ES cells cultured in E8 via precursors of mesendoderm (E8-preME) or 4i ES cells (4i). Values in flowcytometry plots indicate percentage of DMRT1 positive or negative in NANOS3-tdTomato positive cell populations. Scale bar in immunofluorescence image shows 50 μm. The experiment was repeated independently two times with similar results. (**f**) Immunofluorescence of TFAP2C and 5-methylcytosine (5mC) for PGCLCs induced with BSE (d6 + 0) or BSE 3 days followed by RASE (d3 + 6). Scale bar =50 μm. The experiment was repeated independently two times with similar results.

Since BSE induces PGCLC specification but hinders their subsequent development (Fig. 1d), we first induced PGCLCs with BSE and replaced it with Ra on day 2 and with Ra/ActA on day 3, which resulted in a robust expression of CDH5 and DMRT1 in PGCLCs over 8 days (38.6% NANOS3^+^DMRT1^+^ cells) (Extended Data Fig. 2a). The inclusion of SCF and EGF (SE), the PGCLC survival factors^34–36^, together with Ra and ActA, enhanced the induction of DMRT1 and CDH5 (86.9% NANOS3^+^DMRT1^+^ cells on day 8) (Extended Data Fig. 2a). Indeed, the simultaneous addition of Ra and ActA with SCF and EGF (henceforth called RASE) when replacing BSE was most effective for the induction of DMRT1 (Extended Data Fig. 2b). ActA family members, TGF-β and Nodal that also activate SMAD2/3 did not induce DMRT1 (Extended Data Fig. 2c).

**Fig. 2:**
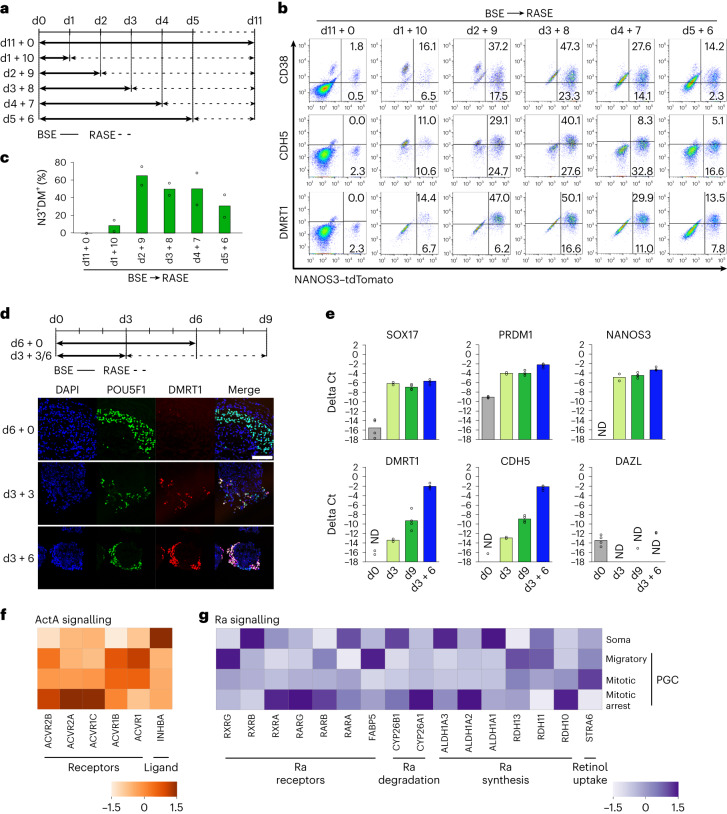
Switching signalling in nascent PGCLCs for further development. **a**,**b**, Flow cytometry of NANOS3–tdTomato against CD38–PerCP–Cy5.5, CDH5–AF647 and DMRT1–mVenus. PGCLCs induced from 4i ES cells for 11 days in the presence of BMP2, SCF and EGF (BSE) or BSE replaced with Ra, ActA, SCF and EGF (RASE) on day (d) 1, 2, 3, 4 or 5 (**a**). Percentage population values of NANOS3-positive and CD38, CDH5 or DMRT1-positive and negative cells in the live cell population (**b**). **c**, Quantification for the percentage of NANOS3 and DMRT1 double-positive cells (%N3^+^DM^+^). Average from two independent experiments. **d**, Immunofluorescence for POU5F1 and DMRT1 co-staining of aggregates induced from 4i ES cells with 6 days of BSE (day 6), or day 3 (day 3 + 3) or 6 (day 3 + 6) replaced with RASE following 3 days of BSE. Scale bar, 100 μm. **e**, RT–qPCR for TNAP-positive 4i ES cells (d0) and day 3 PGCLCs (d3), and for NANOS3–tdTomato reporter-positive cells induced with BSE for 9 days (d9) and 3 days of BSE followed by RASE for 6 days (d3 + 6) from 4i ES cells. Biologically independent experiments, *n* = 4 for d0, d9, d3 + 6 and *n* = 2 for d3, are shown. Delta Ct values are calculated with housekeeping gene *GAPDH*. ND, some of the value(s) not detected. **f**,**g**, Expression of signalling component of ActA (**f**) and Ra (**g**) from in vivo PGC and soma as transcriptome dataset^24^. The colour codes represent *Z*-score. Source data

Nascent PGCLCs induced by BSE were responsive to RASE for CDH5/DMRT1 expression (henceforth DM^+^PGCLCs; Fig. 8b) from day 2 onwards but not on day 1 (Fig. 2a–c), which also occurred in PGCLCs induced from female 4i ES cells and from preME/iMeLCs^11,13^ (Extended Data Fig. 2d,e). DMRT1 protein co-localized with POU5F1 (Fig. 2d) and *SOX17*, *PRDM1*, *DMRT1* and *CDH5* transcripts were detected in PGCLCs induced by BSE followed by RASE (Fig. 2e).

We analysed the available single-cell RNA sequencing (scRNA-seq) of PGCs and gonadal soma^24^ and found expression of ActA (encoded by *INHBA*) in the soma and of the receptors in PGCs (Fig. 2f). Contemporarily, expression of *STRA6*, retinol receptor, and enzymes for Ra synthesis, *RDH11*, *ALDH1A1* and *ALDH1A3*, was present in soma (Fig. 2g). Ra receptor *RAR* family and *FABP5*, which delivers Ra to RARs^37,38^, were expressed in migratory and mitotic PGCs, while *CYP26* enzymes for Ra degradation were low (Fig. 2g). Accordingly, PGCs in vivo can respond to ActA and Ra expressed in surrounding cells that can induce *DMRT1* and *CDH5*.

### DMRT1 can activate DAZL

**Extended Data Fig. 3:**
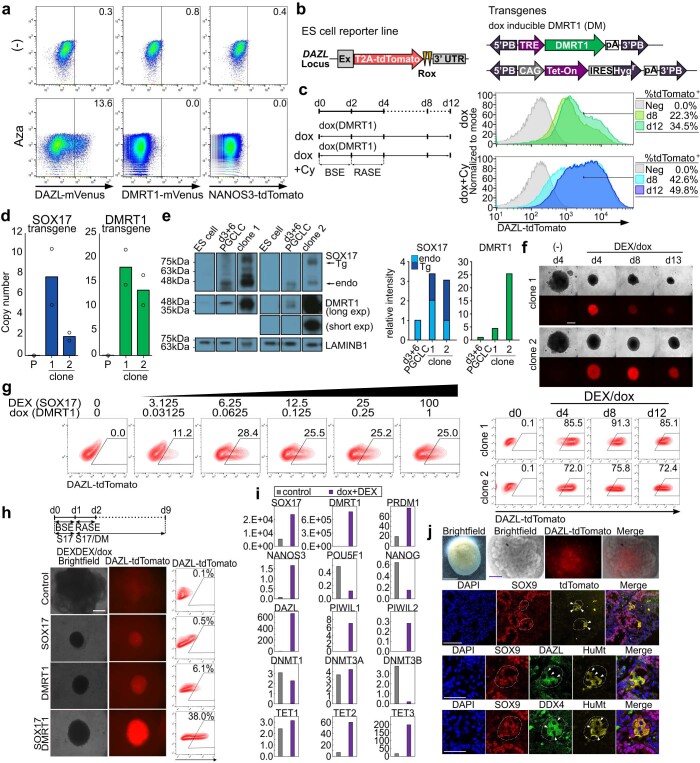
DMRT1 activates DAZL expression. (**a**) Flowcytometry of DAZL-mVenus, DMRT1-mVenus and NANOS3-tdTomato ES cells treated with 5-Aza-2-deoxycytidine (Aza, 5 μM) for 3 days or untreated control (-). % reporter positive cells/live cell population. (**b**) Design for dox-inducible DMRT1 (DM) and DAZL–tdTomato ES cells. (**c**) Flowcytometry of DAZL-tdTomato ES cells with dox-inducible DMRT cultured for 8 or 12 days with doxycycline (dox) with or without cytokines (Cy): BSE followed by RASE. Negative controls (Neg) are Day 0 samples. %DAZL-tdTomato positive cells/live cell population. (**d**) Quantitative PCR for copy number of SOX17 = GR and TRE-DMRT1 plasmids in genomic DNA for clone 1 and 2 cell lines. Biologically independent experiments, n = 2. (**e**) Western blot for SOX17, DMRT1, LMNB1 in nuclear protein extract for transgenic cell lines treated with DEX/dox for 2 days and PGCLC d3 + 6. The experiment was repeated independently two times with similar results. (**f**) Fluorescence images and flowcytometry of DAZL-tdTomato WIS2 ES cells transgenic for clone 1 and 2 with (DEX/dox) or without (-) SOX17/DMRT1 induction treated with BSE followed by RASE as in Fig. 3b. Scale bar =200 μm. (**g**) Flowcytometry of DAZL reporter for dose response to DEX (μM) and dox (μg/ml). (**h**) Fluorescence images and flowcytometry of transgenic DAZL-tdTomato Shef-6 ES cells cultured for 9 days. Scale bar =200 μm. %DAZL-tdTomato positive cells/live cells. The experiment was repeated independently two times with similar results. (**i**) RT-qPCR of transgenic DAZL-tdTomato Shef-6 ES cells induced for 4 days with (dox+DEX) or without (control) dox/DEX. ΔΔCT normalized with RPLP0. The data represent from n = 1 experiment and repeat experiments with independent cell line are shown in Fig. 3c. (**j**) Day 11 testicular organoid reconstituted from E12.5 male mouse gonadal soma28 with DAZL-tdTomato positive PGCLC day 9 induced by DMRT1/SOX17. Brightfield (Top left, Scale bar = 200 μm) and fluorescence (Scale bar = 100 μm) microscopy images. Immunofluorescence of organoids for SOX9, tdTomato, DAZL, DDX4 and human mitochondria (HuMt). Dashed lines show testicular tubules with SOX9 expression. Arrowheads show tdTomato or HuMt positive human PGCLCs. Scale bars: 100 μm (top) and 50 μm (middle/bottom). The experiment was repeated independently two times with similar results. Source data

Expression of DAZL, a DNA methylation-sensitive PGC gene, accompanies a decline in 5mC levels in PGCs, indicating germline commitment^39–42^. A lack of DAZL expression in DM^+^PGCLCs indicates that they are at an earlier stage with substantial overall levels of 5mC (Fig. 2e and Extended Data Fig. 2f). To investigate further, we established ES cell lines with a DAZL reporter (Extended Data Figs. 1c,d and 3b). A DNA-hypomethylating agent, 5-aza-2′-deoxycytidine (Decitabine, Aza), activated the DAZL reporter but not NANOS3 and DMRT1, confirming DNA demethylation-sensitive reporter expression^39–42^ (Extended Data Fig. 3a).

**Fig. 3:**
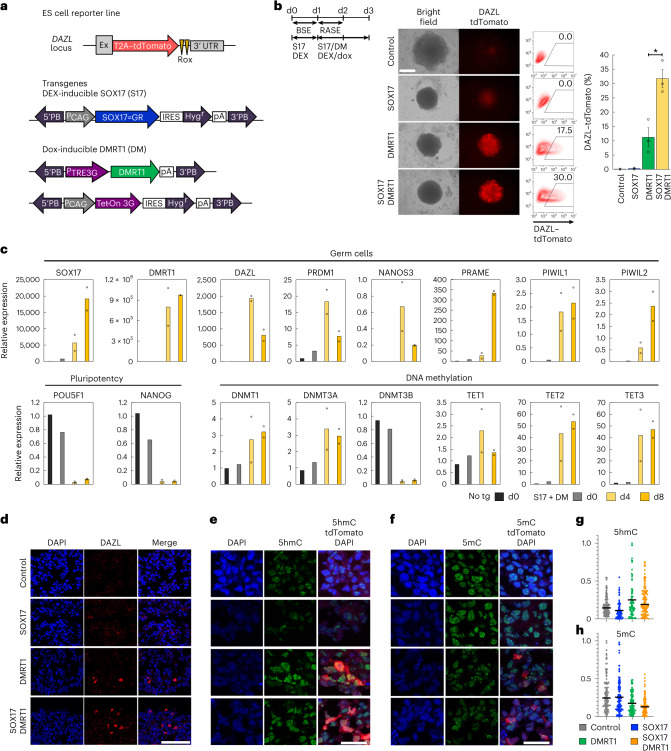
DMRT1 activates DAZL expression. **a**, Expression of DEX-inducible SOX17=GR (S17) and dox-inducible DMRT1 (DM) in DAZL–tdTomato reporter ES cells. **b**, Fluorescence microscope images and flow cytometry of DAZL–tdTomato reporter transgenic male ES cells (WIS2) clone 1 cultured for 3 days with induction of SOX17, DMRT1 or SOX17/DMRT1 in the presence of cytokines: BSE: BMP2/SCF/EGF followed by RASE: Ra/ActA/SCF/EGF. No transgene induction was used as a control. Scale bar, 200 μm. *P = 0.01 calculated using two-tailed *t*-test. Error bars are mean ± standard error of the mean. Biologically independent experiments, *n* = 2 for control, SOX17 and *n* = 3 for DMRT1, SOX17/DMRT1, are shown. **c**, RT–qPCR analysis of DAZL-tdTomato-positive cells induced for 4 and 8 days with the induction of SOX17/DMRT1 transgenes in the presence of cytokines BSE followed by RASE. Values are normalized with housekeeping gene *RPLP0*, and relative values against control samples are presented as mean ± standard deviation. Biologically independent experiments, *n* = 2 for day (d)4 and d8 and the controls (no tg d0, S17 + DM d0) collected independently, are shown. **d**–**f**, Immunofluorescence for DAZL (**d**), co-staining of 5hmC and tdTomato (**e**) and co-staining of 5mC and tdTomato (**f**) of aggregates induced from transgenic ES cell line with 4 day induction of SOX17, DMRT1 or SOX17/DMRT1. Transgenes were not induced for negative controls. Scale bars, 100 μm (**d**) and 30 μm (**e** and **f**). **g**,**h**, Quantification of immunofluorescence for 5hmC-positive (**g**) and 5mC-positive (**h**) cells of day 4 induction of SOX17, DMRT1 or SOX17/DMRT1 transgenes in DAZL–tdTomato ES cell line (Methods). No transgene induction cells were used as controls. Independent cells analysed for 5hmC: control *n* = 140, SOX17 *n* = 80, DMRT1 *n* = 86, SOX17/DMRT1 *n* = 120, and 5mC: control *n* = 113, SOX17 *n* = 130, DMRT1 *n* = 109, SOX17/DMRT1 *n* = 114. The *y* axis indicates value of scale normalization. The median value is indicated by a black bar in the dot plot. The experiment was repeated independently two times with similar results. Source data

Since PGCs in vivo display increasing levels of DMRT1 before DAZL expression (Fig. 1a), we found that the induction of DMRT1 by dox in ES cells over 8–12 days activated DAZL reporter (dox, day 8: 22.3% and day 12: 34.5%) (Extended Data Fig. 3b,c); replacing BSE with RASE enhanced DAZL (dox + Cy, day 8: 42.6% and day 12: 49.8%) (Extended Data Fig. 3c). Co-induction of DMRT1 with SOX17 by DEX showed more efficient activation of DAZL, but SOX17 alone had no effect (Fig. 3a,b,d and Extended Data Fig. 3d–g). DMRT1/SOX17 repressed DNA-methyltransferase *DNMT3B* and induced *TET2* and *TET3*, which is analogous to week 7–9 gonadal PGCs (Fig. 3c)^6^. A female ES cell DAZL reporter line with DMRT1/SOX17 responded similarly (Extended Data Figs. 1b,c and 3d,h,i). DAZL-positive cells showed expression of both early PGC (*PRDM1* and *NANOS3*) and gonadal PGC genes (*PRAME*, *PIWIL1* and *PIWIL2*; Fig. 3c and Extended Data Fig. 3i). Notably, *DMRT1*/*SOX17* suppressed pluripotency genes, *POU5F1* and *NANOG*, as seen in male mitotic arrest (week 9) and female pre-meiotic (week 11) PGCs^6,24^.

We observed a notable increase in 5hmC upon DMRT1, and DMRT1/SOX17 induction, with a reduction in 5mC, but SOX17 alone had no detectable effect (Fig. 3e–h). Altogether DMRT1 alone and with SOX17 is implicated in the epigenetic programming of the human PGCs, and together induced DAZL-positive PGCLCs (hereafter called DZ^+^PGCLCs; Fig. 8b). DZ^+^PGCLCs, when combined with mouse embryonic gonadal cells^28,29^, showed DDX4 expression and colonization of testicular tubules, albeit inconsistently (Extended Data Fig. 3j), which, in principle, indicates their developmental potential.

### Molecular networks towards human germline commitment

**Fig. 4:**
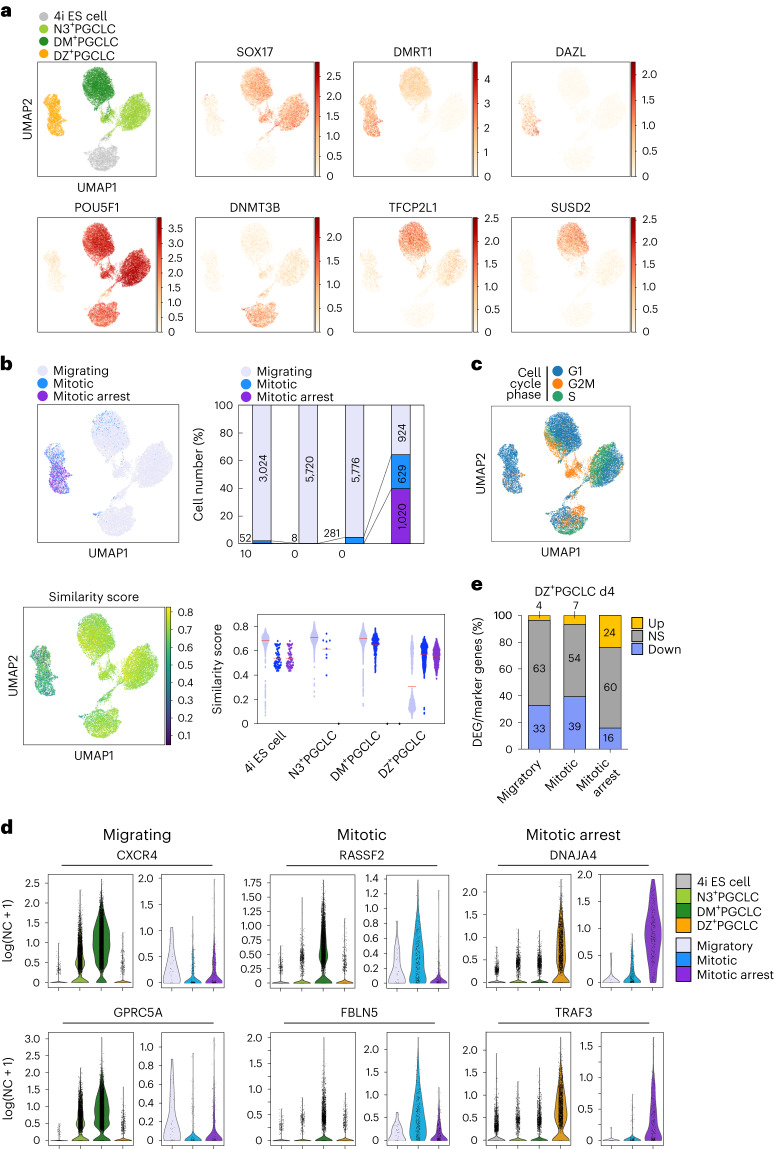
Transcriptome networks for human germline commitment in vitro. **a**, UMAPs of 4i ES cells, N3^+^PGCLC, DM^+^PGCLC and DZ^+^PGCLC day (d)8 scRNA-seq dataset (each from single biological sampling points) batch-corrected with Harmony. Expression of marker genes shown log transformed (normalized count (NC) + 1). SOX17 expression was detected for the endogenous transcripts and not for the chimaeric SOX17=GR transgenes. **b**, Discrete cell type annotations (top) and continuous similarity scores (bottom) transferred with scmap from migratory, mitotic and mitotic arrest PGCs in vivo^24^ onto 4i ES cells, N3^+^PGCLC, DM^+^PGCLC and DZ^+^PGCLC d8 scRNA-seq dataset. UMAPs of 4i ES cells, N3^+^PGCLC, DM^+^PGCLC and DZ^+^PGCLC d8 scRNA-seq dataset batch-corrected with Harmony and labelled by discrete cell type annotations (top left) and continuous similarity scores (bottom left) from migratory, mitotic and mitotic arrest PGCs in vivo^24^. Top right: bar plot of the proportion and exact number of cells in 4i ES cells, N3^+^PGCLC, DM^+^PGCLC and DZ^+^PGCLC d8 scRNA-seq dataset predicted to correspond to either migratory, mitotic or mitotic arrest PGCs in vivo. Bottom right: dot plot for the similarity scores between migratory, mitotic or mitotic arrest PGCs in vivo and 4i ES cells, N3^+^PGCLC, DM^+^PGCLC and DZ^+^PGCLC d8 cells. The mean value is indicated by a red bar in the dot plot. **c**, UMAP of 4i ES cells, N3^+^PGCLC, DM^+^PGCLC and DZ^+^PGCLC d8 scRNA-seq dataset batch-corrected with Harmony and labelled by phase of the cell cycle. **d**, Marker gene expression for in vitro PGCLCs and migratory, mitotic and mitotic arrest PGCs in vivo. NC, normalized count. **e**, Proportion (*y* axis, %) of DEGs from bulk RNA-seq for day 4 DZ^+^PGCLCs against 4i ES cells within marker genes for migratory, mitotic or mitotic arrest PGCs in males. The colour codes represent upregulated (Up, yellow), not significant (NS, grey) and downregulated (Down, blue) DEGs. Number of genes in the categories is indicated in bar graphs.

**Extended Data Fig. 4:**
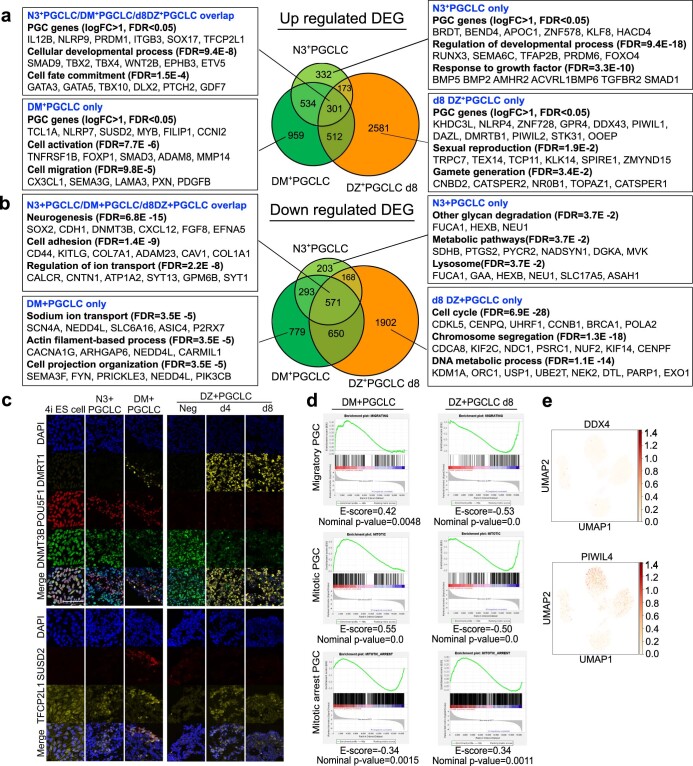
Transcriptome network in in vitro induced PGCLCs. (A and B) Venn diagram for significantly upregulated (Log2 (FC) > 1 and FDR < 0.05) (**a**) and downregulated (Log2 (FC) <-1 and FDR < 0.05) (**b**) DEGs in N3^+^PGCLC, DM^+^PGCLC and DZ^+^PGCLC d8 versus 4i ES cells. The number of genes for each category is indicated in the Venn diagram. Text boxes show representative PGC genes and related Gene Ontology (GO) biological processes terms significantly enriched with FDR values. The data represents an integration of two biological replicates. FC: Fold Changes. (**c**) Immunofluorescence for marker genes in 4i ES cells, N3^+^PGCLC, DM^+^PGCLC and DZ^+^PGCLC (day 4 (d4), day 8 (d8), no DEX/dox control for 8 days (Neg)). TFCP2L1 for 4i ES cells shows high background signal. Scale bar: 100 μm. The experiment was repeated independently two times with similar results. **(d**) Gene set enrichment analysis (GSEA) for DM^+^PGCLC and DZ^+^PGCLC d8 versus 4i ES cells based on RNA-seq against marker genes for migratory, mitotic, and mitotic arrest PGCs. E-score (enrichment score) and nominal p-value are indicated. The p-values for the GSEA test statistics are calculated by permutation. The data represents an integration of two biological replicates. (**e**) UMAPs of 4i ES cells, N3^+^PGCLC, DM^+^PGCLC and DZ^+^PGCLC d8 scRNA-seq dataset (each from single biological sampling points) batch-corrected with Harmony. Expression of *DDX4* and *PIWIL4* shown in log transformed (normalized count (NC) + 1).

Next, we performed bulk and scRNA-seq for 4i ES cells, NANOS3 (N3)^+^PGCLC (day 3), DM^+^PGCLCs and DZ^+^PGCLCs (day 4 and/or day 8) (Fig. 4). Differentially expressed gene (DEG) analysis of bulk RNA sequencing (RNA-seq) revealed 301 upregulated genes in N3^+^PGCLC, DM^+^PGCLCs and DZ^+^PGCLCs, including ‘PGC genes’ *SOX17*, *PRDM1*, *TFCP2L1* and *NLRP9* (Methods), ‘cellular developmental process’ and ‘cell fate commitment’ by Gene Ontology (GO) (Extended Data Fig. 4a). Upregulated PGC genes include *BRDT*, *BEND4*, *KLF8* and *HDAC4* for N3^+^PGCLCs, while DM^+^PGCLCs showed expression of *TCL1A* and *SUSD2*, markers for migratory and mitotic PGCs, respectively (Methods)^24,28^, and GO terms ‘cell activation’ and ‘cell migration’ (Extended Data Fig. 4a). Mitotic arrest PGC markers *PIWIL1* and *PIWIL2*, as well as GO terms ‘sexual reproduction’ and ‘gamete generation’, were upregulated explicitly in DZ^+^PGCLCs. The commonly downregulated genes related to neurogenesis, cell adhesion and ion transport were identified. For N3^+^PGCLCs, we found glycan degradation and metabolic pathways, and for DM^+^PGCLCs we observed sodium ion transport and actin filament-based process. For DZ^+^PGCLCs, we found cell cycle and chromosome segregation among significantly downregulated DEGs (Extended Data Fig. 4b).

With scRNA-seq analysis, we generated Uniform Manifold Approximation and Projection (UMAP) with Harmony integration (Methods), revealing four clusters (Fig. 4a); *SOX17*, *DMRT1*, *DAZL*, *POU5F1*, *DNMT3B*, *TFCP2L1* and *SUSD2* were enriched in specific clusters in the UMAPs (Fig. 4a and Extended Data Fig. 4c). Label transfer from in vivo male PGCs migratory/mitotic/mitotic arrest^24^ to PGCLCs in the UMAP (Fig. 4b) showed 4i ES cells and N3^+^PGCLCs were labelled mostly with migratory PGCs, while DM^+^PGCLCs exhibited migratory PGC labels for 5776 cells (similarity score 0.70/1.0) and a subpopulation of mitotic PGC labels for 281 cells (similarity score 0.6/1.0) (Fig. 4b). By contrast, for DZ^+^PGCLCs, 924 cells were labelled with migratory, 629 cells with mitotic and 1,020 cells with mitotic arrest; the latter two had a higher similarity score >0.55 compared with the score for migratory of 0.31 (Fig. 4b). DZ^+^PGCLCs also indicated mitotic arrest features due to lacking G2/M and S phases (Methods) (Fig. 4c). Specific marker genes for each in vitro induced group, N3^+^, DM^+^ and DZ^+^ PGCLCs, were reflected in the expression pattern for in vivo PGC developmental phases (Fig. 4d). Consistently, gene set enrichment analysis (GSEA) using bulk RNA-seq revealed significant upregulation of migratory/mitotic PGC genes in DM^+^PGCLCs, and significant enrichment of mitotic arrest genes in DZ^+^PGCLCs (Extended Data Fig. 4d). While some mitotic arrest PGC markers, such as *DDX4* and *PIWIL4*, were undetectable (Extended Data Fig. 4e), 24% of mitotic arrest PGC markers were upregulated DEGs in DZ^+^PGCLCs (Fig. 4e). Interestingly, 33% of the migratory and 39% of the mitotic PGC markers were downregulated in DZ^+^PGCLCs (Fig. 4e). A heat map provides an overall view of the key transcriptional differences between the three key stages of transitions from nascent to advanced PGCLCs (Fig. 8a). Overall, the PGCLC progression reflects PGC development in vivo towards germline commitment.

### DMRT1 targets for human germline commitment

**Fig. 5:**
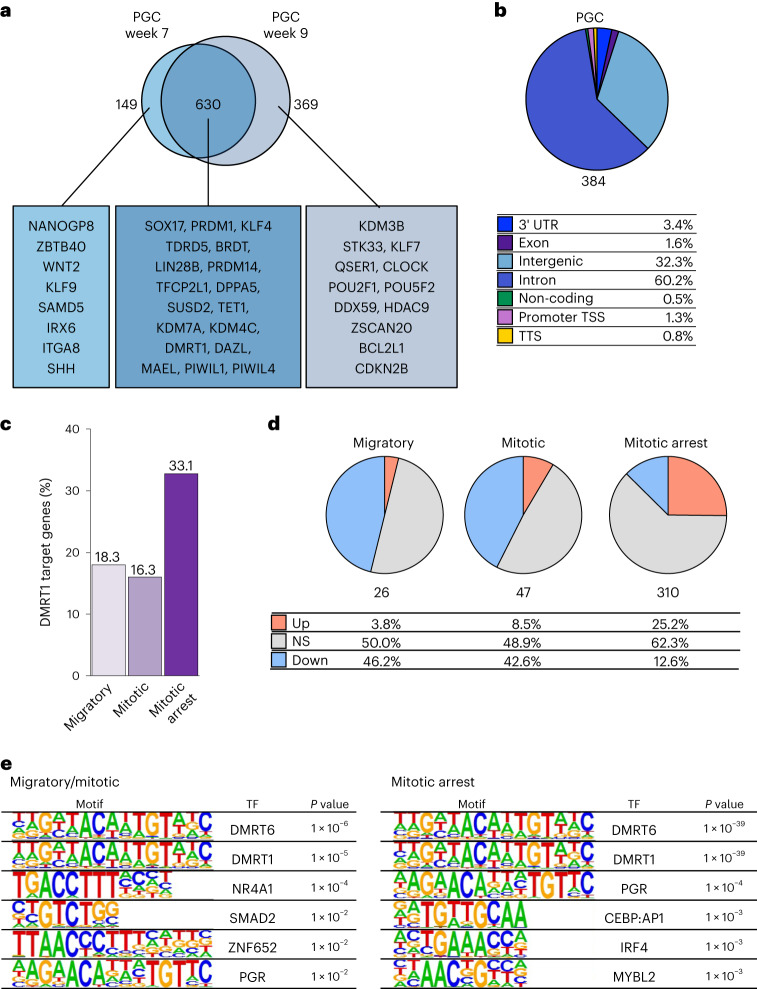
DMRT1 targets for foetal germline development. **a**, Venn diagram for comparing week 7 and week 9 upregulated PGC DEGs that are targets of DMRT1 identified by C&R. Text boxes indicate representative DMRT1 target PGC genes shared or unique in week 7 and week 9 male PGCs. **b**, Pie chart presents the distribution of genomic features for DMRT peaks (*n* = 384) associated with PGC genes. The percentage (%) of the target peaks is shown for each category. **c**, Proportion (*y* axis, %) of DMRT1 targets in d4 DZ^+^PGCLCs within the marker genes for migratory (18%; 26/142 genes), mitotic (16%: 47/288) or mitotic arrest (33%: 310/937 genes) PGCs. **d**, Proportion of DMRT1 targets for upregulated (Up, orange), not significant (NS, grey) and downregulated (Down, blue) genes for migratory, mitotic or mitotic arrest PGC in d4 DZ^+^PGCLCs versus 4i ES cells. **e**, Topmost commonly known motifs enriched in DMRT1 peaks. Left: motifs enriched in DMRT1 peaks associated with marker genes for migratory and mitotic male PGCs. Right: motifs enriched in DMRT1 peaks associated with marker genes for mitotic arrest male PGCs. Enrichment *P* values are calculated using binomial distributions. The data in **a**–**e** represent an integration of three biological replicates.

**Extended Data Fig. 5:**
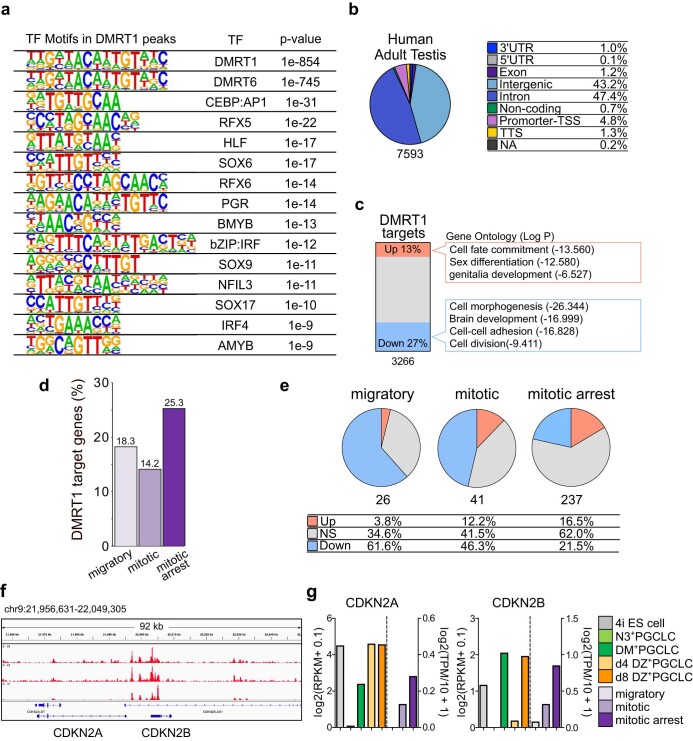
DMRT1 targets for human fetal germ cell development. (**a**) Topmost commonly known transcription factor (TF) motifs enriched in all DMRT1 peaks in d4 DZ^+^PGCLCs identified by C&R using Homer (cumulative binomial distributions). The data represents an integration of three biological replicates. (**b**) Distribution of genomic features with DMRT1 peaks identified in human adult testis^46^. Total number of peaks and proportion (%) of genomic features in all the peaks are indicated. (**c**) The bar plot represents the % of overlap between DMRT1 target genes and upregulated (Log2 Fold Changes (FC) > 1 and FDR < 0.05) and downregulated (Log2 (FC) < -1 and FDR < 0.05) genes in d8 DZ ^+^ PGCLC versus 4i ES cells. Text boxes show the related Gene Ontology (GO) biological processes terms significantly enriched. P-value is calculated based on a cumulative hypergeometric distribution. The data represents an integration of two biological replicates. (**d**) Proportion (y-axis, %) of DMRT1 targets within the marker genes for migratory (26/142 genes: 18%), mitotic (41/288 genes: 14%) or mitotic arrest PGCs (237/937 genes: 25%) in d8 DZ+PGCLCs. The data represents an integration of two biological replicates. (**e)** Proportion of DMRT1 targets for upregulated (Up, orange), not significant (NS, grey), and downregulated (Down, blue) genes for migratory, mitotic or mitotic arrest PGC in d8 DZ^+^PGCLCs versus 4i ESCs. The data represents an integration of two biological replicates. (**f**) DMRT1 C&R peaks at CDKN2A and CDKN2B genomic locus in d4 DZ ^+^ PGCLC. Technical triplicates are shown. (**g**) Expression of CDKN2A and CDKN2B in 4i ES cells, PGCLCs and PGCs from RNA-seq. Y-axes are log2 (RPKM + 0.1), left and log2 (TPM/10 + 1), right. Bar plot represents the mean (4i ES cells and PGCLCs, n = 2 biological replicates; migratory, 37 cells; mitotic, 332 cells; mitotic arrest, 309 cells).

For mechanistic insights concerning DMRT1 and genomic targets, we performed CUT&RUN (C&R)^43–45^ for DZ^+^PGCLCs (on days 4 and 8), which confirmed DMRT1 binding specificity (Extended Data Fig. 5a). Of the 1,148/4,171 protein-coding genes, PGC genes in day 4 DZ^+^PGCLC were closest to the DMRT1 peaks (henceforth DMRT1 targets). The 630 commonly expressed genes in week 7 and week 9 PGCs include *SOX17*, *PRDM1* and *KLF4*; pluripotency genes, *PRDM14*, *TFCP2L1* and *SUSD2*; epigenetic regulators, *TET1*, *KDM7A* and *KDM4C*; and gonadal PGC genes, *DAZL*, *MAEL*, *PIWIL1* and *PIWIL4* (Fig. 5a). Of these, 149 PGC genes, including *NANOGP8* and *KLF9*, were specific to week 7, while 369 genes, such as *STK33*, *QSER1* and *DDX59*, were specific to week 9 PGCs (Fig. 5a). Over 60% of the DMRT1 peaks for PGC genes were at introns, 32% were intergenic and 1.3% were at promoter transcriptional start sites (TSSs) (Fig. 5b). Intronic and intergenic binding by DMRT1 (47.4% and 43.2%, respectively) was also observed in human testis (Extended Data Fig. 5b)^46^.

Combining the C&R with bulk RNA-seq for day 8 DZ^+^PGCLCs revealed that 13% of DMRT1 target genes were upregulated with GO terms ‘Cell fate commitment’ and ‘sex differentiation’, while 27% of the target genes were downregulated with GO terms ‘Cell morphogenesis’ and ‘Brain development’ (Extended Data Fig. 5c). We found more DMRT1 targets for mitotic arrest PGC genes when compared with that for migratory and mitotic PGC genes in DZ^+^PGCLCs (Fig. 5c and Extended Data Fig. 5d). More downregulated target genes were for migratory and mitotic PGCs, than for mitotic arrest PGCs. In contrast, more DMRT1 targets for mitotic arrest genes were upregulated compared with migratory and mitotic genes in DZ^+^PGCLCs (Fig. 5d and Extended Data Fig. 5e). DMRT1 targets, *CDKN2A* and *CDKN2B* cell cycle inhibitors, were upregulated in DZ^+^PGCLCs and mitotic arrest PGCs in vivo (Extended Data Fig. 5f,g). Significant enrichment of NR4A1, SMAD2 and ZNF652 binding motifs was found at DMRT1 peaks for migratory/mitotic PGCs genes, while significant enrichment of PGR (progesterone receptor), CEBP:AP1, IRF4 and MYBL2 motifs was enriched for mitotic arrest PGC genes (Fig. 5e), suggesting distinct regulation by DMRT1 for gene suppression in migratory/mitotic PGCs, and gene induction in mitotic arrest PGCs, potentially with a stage-specific binding partner(s).

### DNA modification dynamics involving DMRT1

**Fig. 6:**
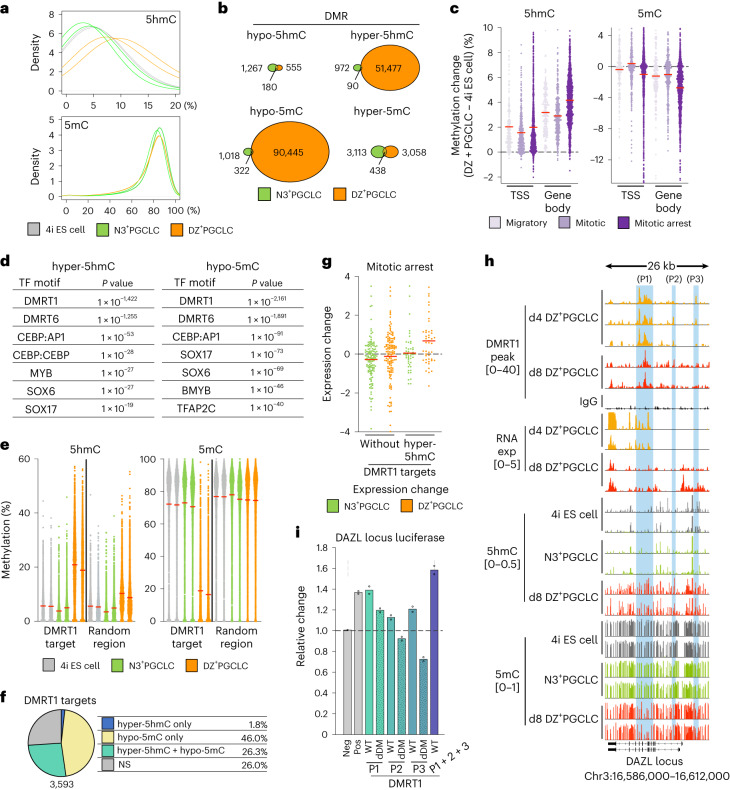
DNA methylation profiling and DMRT1 in early human germline. **a**, Percentage of 1 kb tiles (≥3 CpGs, covered >5 times) methylation values across 4i ES cells, N3^+^PGCLC and day (d)8 DZ^+^PGCLC methylome. The density plots (bandwidth 0.05) show the distribution of 5hmC or 5mC level across the genome (5hmC, *n* = 2,657,811 tiles; 5mC, *n* = 2,622,626 tiles). Two biological replicates of each cell type are shown. **b**, Venn diagrams for DMRs in d8 DZ^+^PGCLC/4i ES cells and N3^+^PGCLC/4i ES cells. The data represent an integration of two biological replicates. **c**, DNA methylation of d8 DZ^+^PGCLC/4i ES cells at approximately −2 to 0 kb TSS and gene body regions for 142 migratory, 288 mitotic and 937 mitotic arrest PGC genes. The mean value is indicated by a red bar in the dot plot. The data represent an integration of two biological replicates. Dashed horizontal line indicates no change in % methylation. **d**, Topmost TF motifs enriched in DMRs for hyper-5hmC (51,567 DMRs) and hypo-5mC (90,767 DMRs) in d8 DZ^+^PGCLCs/4i ES cells. *P* values by binomial distributions. **e**, DNA methylation levels of DMRT1 peaks (average length 369 bp, ≥3 CpGs, covered >5 times in all samples) and random regions (*n* = 3,351). The mean value is indicated by a red bar in the dot plot. Two biological replicates of each cell type are shown. **f**, Percentage of overlap between DMRs and DMRT1 peaks (≥3 CpGs, covered >5 times in all samples). The data represent an integration of two biological replicates. **g**, Expression changes for d8 DZ^+^PGCLCs/4i ES cells of mitotic arrest PGC genes with DMRT1 peaks with (*n* = 46 genes) or without (*n* = 131 genes) hyper-5hmC DMRs in their intronic regions. The mean value is indicated by a line in the dot plot. Dashed horizontal line marks no change in expression. **h**, Visualization for DMRT1 C&R peaks, bulk RNA-seq, 5hmC and 5mC at *DAZL* genomic locus. **i**, Luciferase assay for DMRT1 binding sites at *DAZL*. Peak 1 (P1), 2 (P2) and 3 (P3) indicated in **h** and the deletion of the DMRT1 motif were examined with DMRT1 induction. Empty luciferase vector (Neg: negative control) and that with SV40 enhancer (Pos: positive control). Relative values against Neg. Biologically independent experiments, *n* = 2. Dashed horizontal line indicates the empty luciferase vector signal activity (Neg sample), set to 1. Source data

Since the upregulation of 5hmC and downregulation of 5mC was detected by immunofluorescence in DZ^+^PGCLCs (Fig. 3e–h) as in PGCs in vivo^6^, we performed chemical-assisted pyridine borane sequencing plus (CAPS+) for 5hmC, and TET-assisted pyridine borane sequencing with β-glucosyltransferase blocking (TAPSβ) for 5mC to generate single-base-resolution profiling^47,48^ in DZ^+^PGCLCs, 4i ES cells and N3^+^PGCLCs (Fig. 6).

**Extended Data Fig. 6:**
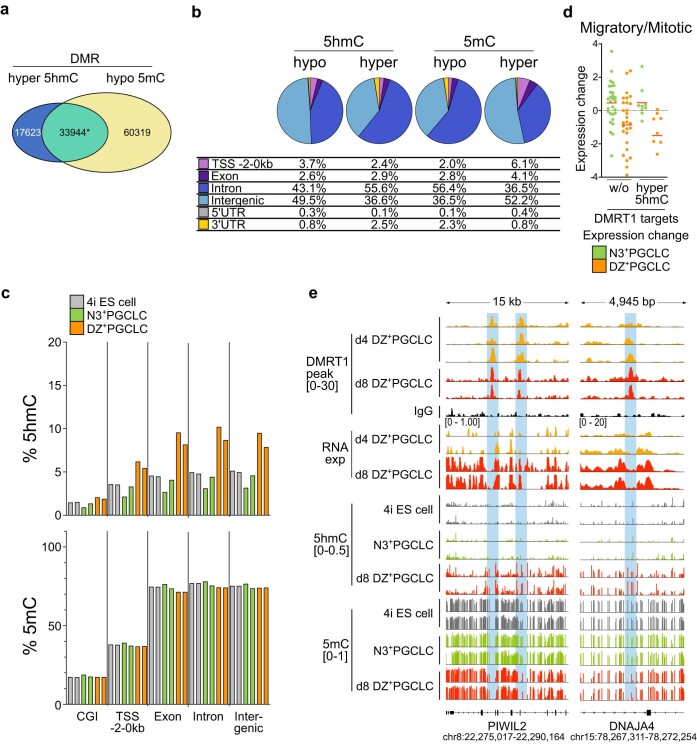
5hmC and 5mC profiling in DZ+PGCLCs. (**a**) Venn diagrams showing the overlap of DMRs for hyper 5hmC and hypo 5mC in d8 DZ^+^PGCLCs compared to 4i ES cells. Number of DMRs are indicated. *p-value = e-104768 for the fisher’s extract test, two-tailed. The data represents an integration of two biological replicates. (**b**) Pie chart presents the distribution of genomic features for DMRs. The data represents an integration of two biological replicates. (**c**) Bar plots showing the methylation levels of different genomic features (CGI, n = 25612; TSS-2 ~ 0 kb, n = 26418; Exon, n = 207932; Intron, n = 149208; Intergenic, n = 17061). Two biological replicates of each cell type are shown. (**d**) Dot plot showing the expression changes (d8 DZ^+^PGCLCs-4i ES cells) of marker genes for migratory/mitotic PGCs which have DMRT1 peaks with (n = 9 genes) or without (n = 29 genes) DMRs for hyper 5hmC in their intronic regions. The mean value is indicated by a red bar in the dot plot. The data represents an integration of two biological replicates. (e) DMRT1 C&R peaks, bulk RNA-seq, 5hmC and 5mC at PIWIL2 and DNAJA4 locus.

The global 5hmC levels of 10.1% in DZ^+^PGCLCs compared with 5.5% for 4i ES cells and 4.2% for N3^+^PGCLCs (Fig. 6a), while 5mC levels in DZ^+^PGCLCs, 4i ES cells and N3^+^PGCLCs were largely similar at ~76–79% (Fig. 6a). Strikingly, however, we identified notable overlapping hyper-5hmC and hypo-5mC differentially methylated regions (DMRs) in DZ^+^PGCLC (Fig. 6b and Extended Data Fig. 6a), which indicates a dynamic enrichment of 5hmC accompanied by a locus-specific loss of 5mC during differentiation. The hyper-5hmC and hypo-5mC mostly occurred at the DMRT1-bound regions in introns and the intergenic regions (Fig. 5b and Extended Data Figs. 5b and 6b). We observed further enrichment of 5hmC and loss of 5mC at the gene body of mitotic arrest genes compared with migratory and mitotic PGC genes, but not in TSS regions (Fig. 6c). Notably, the transcription factor (TF) binding motifs at the DMRs identified DMRT1 for both hyper-5hmC and hypo-5mC (Fig. 6d). SOX17 was also one of the motifs significantly enriched for both DMRs (Fig. 6d). Consistently, further enrichment of 5hmC and marked depletion of 5mC in DZ^+^PGCLCs was found at DMRT1 binding regions identified by C&R, compared with random regions (Fig. 6e and Extended Data Fig. 6c). A substantial proportion of DMRT1 targets overlapped with hyper-5hmC, hypo-5mC or both (Fig. 6f), suggesting a correlation between DMRT1 genomic binding and DNA methylation changes. Integrative analysis for the methylome, C&R and bulk RNA-seq revealed notable upregulation of DMRT1 targeted mitotic arrest PGC genes with hyper-5hmC in DZ^+^PGCLCs (Fig. 6g). Conversely, downregulation of DMRT1 targeted migratory and mitotic PGC genes occurred independently of the hyper-5hmC in DZ^+^PGCLCs (Extended Data Fig. 6d), implying mitotic arrest stage-specific regulation of gene expression by DMRT1 and 5hmC. Genomic loci for upregulated genes in DZ^+^PGCLCs, such as *DAZL*, *PIWIL2* and *DNAJA4*, displayed multiple sites with hyper-5hmC/hypo-5mC, which co-localized with DMRT1 peaks (Fig. 6h and Extended Data Fig. 6e). Luciferase assay for those regions at the *DAZL* locus with DMRT1 binding motifs showed transcriptional activation in response to DMRT1 (Fig. 6i). Accordingly, DMRT1 binding and consequent DNA methylation changes can regulate stage-specific gene activation. The regulatory mechanism could also involve histone modifications such as the acquisition of H3K36me3 and loss of H3K27me3 with the 5hmC enrichment in gene bodies^49–51^, which merits further investigation.

### DMRT1 and genomic repeat elements in germline

**Fig. 7:**
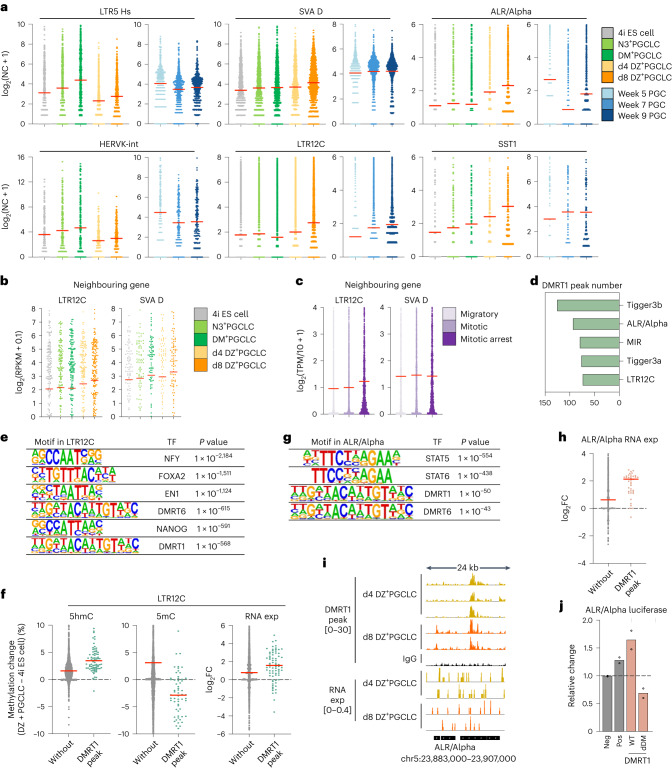
Regulation of genomic repeat elements and DMRT1 in early human germline. **a**, Expression of genomic repeats. The mean value is indicated by a red bar in the dot plot. NC, normalized count. **b**, Expression of genes nearest LTR12C (*n* = 206 genes) and SVA_D (*n* = 95 genes). The mean value is indicated by a red bar in the dot plot. **c**, Expression of genes nearest LTR12C (*n* = 1,093 genes) or SVA_D (*n* = 784 genes) in migratory/mitotic/mitotic arrest male PGCs. The mean value is indicated by a red bar in the dot plot. **d**, Top repeat subfamilies with DMRT1 peaks in day (d)8 DZ^+^PGCLCs. **e**, Topmost TF motifs enriched in LTR12C (*n* = 2,816). *P* values by binomial distributions. **f**, Methylation (5hmC/5mC) and RNA expression (exp) changes of LTR12C with (*n* = 77) or without (*n* = 2,739) DMRT1 peaks in d8 DZ^+^PGCLCs/4i ES cells. The mean value is indicated by a red bar in the dot plot. Dashed horizontal line indicates no change in % methylation (5hmC and 5mC) or RNA expression levels (RNA exp). FC, fold changes. **g**, Topmost TF motifs at ALR/Alpha (*n* = 1,912). *P* values by binomial distributions. **h**, Expression changes of ALR/Alpha with (*n* = 41) or without (*n* = 1,871) DMRT1 peaks in d8 DZ^+^PGCLCs/4i ES cells. The mean value is indicated by a red bar in the dot plot. Dashed horizontal line marks no change in ALR/Alpha RNA expression. FC, fold changes. The data in **a**–**h** represent an integration of two biological replicates. **i**, DMRT1 CR peaks and RNA-seq at ALR/Alpha. **j**, Luciferase assay for ALR/Alpha and that with DMRT1 motif deletion (dDM) after DMRT1 induction. Empty luciferase vector (Neg: negative control) and that with SV40 enhancer (Pos: positive control). Relative values against Neg. Independent experiments, *n* = 2. Dashed horizontal line indicates the empty luciferase vector signal activity (Neg sample), set to 1. Source data

**Extended Data Fig. 7:**
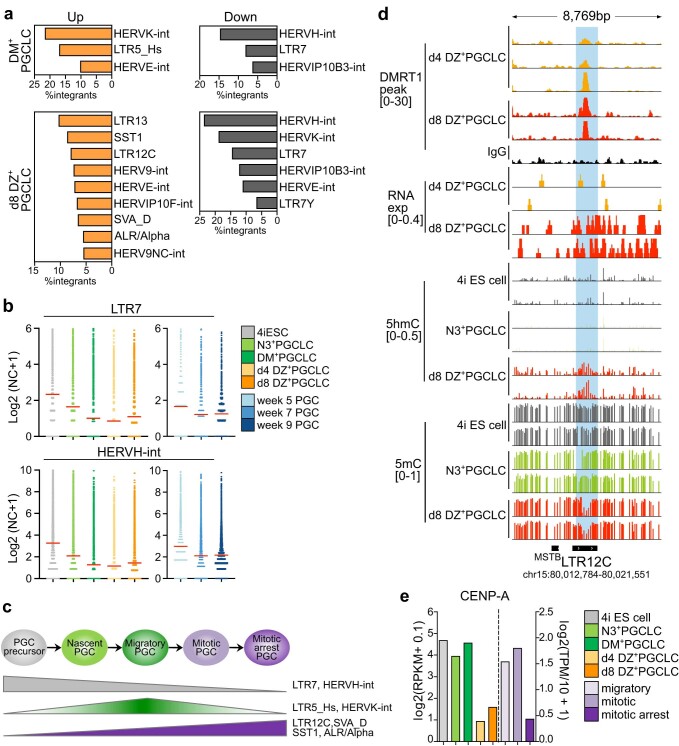
Regulation of genomic repeat elements and DMRT1 in human early germline. (**a**) Differentially expressed repeats at |log Fold Changes (FC)|> 1 and FDR ≤ 0.05 in DM+ PGCLCs or d8 DZ+ PGCLCs versus 4i ES cells. The bar charts show the top subfamilies (> 200 total integrants in the human genome) with the percentage of differentially expressed integrants over total integrants. The data represents an integration of two biological replicates. (**b**) Dot plots for expression of genomic repeat subfamilies with read count≥1 in the sample set. Y-axis is log2 transformed (normalized count (NC) + 1). The mean value is indicated by a red bar in the dot plot. The data represents an integration of two biological replicates. (**c**) Schematic model for expression dynamics of repeat elements during human PGC development. (**d**) DMRT1 C&R peaks, bulk RNA-seq, 5hmC and 5mC at LTR12C in chromosome 15. (**e)** Expression of CENPA in 4iES cells, PGCLCs and PGCs from RNA-seq. Y-axes are log2 (RPKM + 0.1), left and log2 (TPM/10 + 1), right. Bar plot represents the mean (4iES cells and PGCLCs, n = 2 biological replicates; migratory, 37 cells; mitotic, 332 cells; mitotic arrest, 309 cells).

Expression of genomic repeat elements reflects distinct cell-type-specific features in human germline development^28^. We found downregulation of LTR7 and HERVH-int in DM^+^PGCLCs and DZ^+^PGCLCs, compared with 4i ES cells (Extended Data Fig. 7a–c). There was upregulation of LTR5_Hs and HERVK-int in DM^+^PGCLCs as in week 5 PGCs (Fig. 7a and Extended Data Fig. 7a,c), whereas SVA_D, LTR12C, ALR/Alpha (a centromeric satellite) and SST1 displayed higher expression in DZ^+^PGCLCs and week 9 PGCs (Fig. 7a and Extended Data Figs. 7a,c), where mitotic arrest PGCs start to be detectable^24^.

We found notable expression of the hominoid-specific LTR12C elements^52^ (Fig. 7a), together with the neighbouring genes in day 8 DZ^+^PGCLCs (Fig. 7b), as in mitotic arrest PGCs in vivo (Fig. 7c). However, genes proximate to SVA_D (another evolutionarily young transposon) did not show transcriptional activation^53^ (Fig. 7b,c). There are 75 DMRT1 binding sites at LTR12C in DZ^+^PGCLCs based on C&R analysis (Fig. 7d,e), and LTR12C bound by DMRT1 had higher 5hmC and lower 5mC consistent with LTR12C expression, compared with those without DMRT1 binding (Fig. 7f and Extended Data Fig. 7d). Accordingly, we reveal a hominoid-specific role of DMRT1 as well as a regulatory role of LTR12C. We also detected 95 DMRT1 peaks at ALR/Alpha centromeric repeats, which showed higher expression in DZ^+^PGCLC (Fig. 7d,g–i). Luciferase assays confirmed transcriptional activation of ALR/Alpha in response to DMRT1 (Fig. 7j). Together with decreased expression of *CENPA*, a component of the centromere complex^54^ in mitotic arrest PGCs and DZ^+^PGCLCs (Extended Data Fig. 7e), DMRT1 is potentially involved in regulating centromere to control the cell cycle in the human male germline. Our study provides a robust model for mechanistic studies on the role of DMRT1 concerning non-coding genomic regions.

## Discussion

**Fig. 8:**
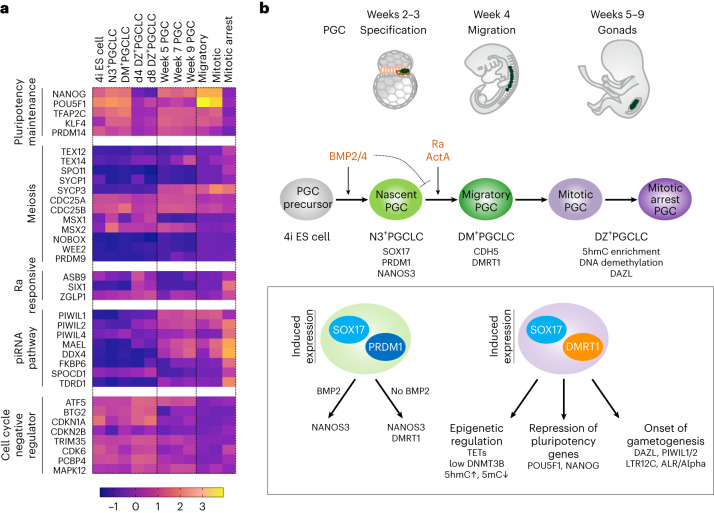
Molecular regulation of human germline commitment. **a**, Heat map presents gene expression *Z*-score of the sample set indicated for enriched genes in 4i ES cells (4i), N3^+^PGCLCs (N3), DM^+^PGCLCs (DM), DZ^+^PGCLCs (day (d)4, 4DZ; d8, 8DZ), week 5/7/9 PGCs, migratory, mitotic and mitotic arrest PGCs. piRNA, PIWI-interacting RNA. The data show an integration of two biological replicates. **b**, Summary scheme for the molecular regulation of human germline commitment. Human PGC specification (approximately weeks 2–3) from the precursors (4i ES cells) in response to BMP2 or BMP4, which induces expression of SOX17 and PRDM1, followed by NANOS3 (N3^+^PGCLCs). Further development occurs by switching the signalling from BMP to Ra and ActA, marked by the expression of CDH5 and DMRT1 (DM^+^PGCLCs), characteristics for migratory/mitotic PGCs (approximately weeks 4–5). Migratory PGCs undergo progressive DNA demethylation through transient 5hmC enrichment, followed by the expression of DAZL (DZ^+^PGCLCs), indicating germline commitment characterized as mitotic arrest status in males (approximately weeks 5–9). Induced expression of SOX17 and PRDM1 can induce *NANOS3*, but the expression of DMRT1 occurs only in the absence of BMP2, suggesting the suppressive effect on DMRT1. Induced expression of DMRT1 and SOX17 modulates the epigenetic programme, including the enrichment of 5hmC and loss of 5mC directly bound by DMRT1, and the potential regulators, *TETs* and *DNMT3B*. DMRT1 is also involved in the suppression of pluripotency genes and induction of later PGC programme, *DAZL*, *PIWIL1* and *PIWIL2*, as well as specific repeat elements, LTR12C and ALR/Alpha, collectively the hallmarks of germline commitment towards the onset of gametogenesis.

We reveal a critical role of DMRT1 in epigenetic resetting and transcription regulation during human germ cell lineage commitment. Specification of PGCs (approximately weeks 2–3) is followed by a multi-step process towards irreversible commitment and gain of competence for gametogenesis^25,42,55,56^. The functional role of DMRT1 in vivo and in vitro coincides with the gain of 5hmC and loss of 5mC, which is exemplified by the expression of DAZL, a DNA methylation-sensitive gene (Fig. 8b)^6,24^. DMRT1 expression commences in migrating male and female PGCs, with a progressive increase in DAZL, which marks germline commitment; later, expression of DMRT1 in germ cells is restricted to males^27,57–60^. Our in vitro model enables mechanistic studies that are hampered by limited access to week 2–6 human embryos.

A transient BMP signal is essential and sufficient for PGCLC specification following the induction of SOX17 and PRDM1 (refs. ^11,14,61^). However, the sustained presence of BMP interferes with DMRT1 expression in PGCLCs; replacing it with ActA and Ra after specification induces DMRT1 and CDH5, respectively, marking the progression of PGCLCs to the migratory stage. The post-specification signalling system comprising ActA and Ra is present in somatic tissues along the migratory route surrounding the PGCs in vivo (Fig. 2f,g). ActA is also crucial for differentiating mesendoderm, yolk sac endoderm and definitive endoderm forming hindgut later^62–64^, and Ra regulates anterior–posterior patterning with the gradient expression^65,66^. Activin and Ra have a role in migrating PGCs in mice^67–70^. CDH5, a homophilic adhesion molecule^71^, may promote interactions between PGCs and surrounding tissues; notably, CDH5 is not detectable in mouse or cynomolgus monkey PGCs^17^.

DMRT1 induces the expression of DNA methylation-sensitive PGC genes, *PIWIL2* and *DAZL*^39–41^ through converting 5mC to 5hmC in DZ^+^PGCLCs, which is evident in PGCs in vivo^6^. DMRT1 also causes the downregulation of *DNMT3B* (but not *DNMT3A*) and upregulation of *TET2* (but not *TET1*). In mice, DNMT3B is required for DNA methylation at gene bodies, while DNMT3A is preferentially recruited to enhancers/promoters^72–76^. Tet2 depletion in mice reduces 5hmC in gene bodies, while Tet1 depletion decreases 5hmC at promoters^77^, which is also reflected in the gain of 5hmC/loss of 5mC at gene bodies in DZ^+^PGCLCs. We observed a localized enhancement of 5hmC and a decrease in 5mC with DMRT1 binding. Similarly, localized DNA demethylation occurs by recruitment of TET1 and TET2 by FOXA1, a pioneer TF, to its binding sites^78^. The direct targets of DMRT1, *TET1* and *QSER1*, protect DNA hypomethylated regions from de novo methylation^79^. Further mechanistic studies are required to elucidate the molecular regulation for the site-specific 5hmC/5mC by DMRT1. SOX17 and PRDM1 also contribute to the epigenetic reprogramming through the upregulation of *TET1*/*TET2*, histone demethylases and the repression of *DNMT3B*^6,61^. Unlike in mice, DAZL expression commences in migrating and proliferating human PGCs^27^, which is incompatible with the onset of meiotic gene expression; these aspects may be regulated differently in humans. DMRT1 binding to hominoid-specific LTR12C with the 5hmC/5mC epigenetic changes also reflect species differences; the upregulation of some neighbouring genes, including mitotic arrest genes, suggests their regulatory role in human PGCs. Activation of SVA_D, another evolutionarily young transposable element^53^, occurred without detectable upregulation of neighbouring genes. The evolution of the transcriptional regulation by DMRT1 through unique repeat elements for germ cell development merits further investigation.

DMRT1 expression occurs both in PGCs and gonadal somatic cells, but global DNA demethylation occurs only in PGCs^6–9,24^. In this context, SOX17 expression is restricted to germ cells, suggesting the combination of SOX17/DMRT1 contributes to DNA demethylation as we found both motifs at hyper-5hmC/hypo-5mC in DZ^+^PGCLCs. Tumours induced by the Yamanaka pluripotency factors (OSKM) exhibit DNA hypomethylation and DAZL expression through DMRT1 (ref. ^80^), suggesting that a combinatory effect of DMRT1 with other factors, such as pluripotency genes, enables regulation of DNA methylation for germline commitment. Dmrt1 is also present in rodent germline^58,59^ but without Sox17 (refs. ^13,19–21^), indicating potential mechanistic and functional species differences^81^. The mitotic arrest of PGC/PGCLCs might also, in part, be induced by DMRT1 following the migratory and mitotic phase of PGCs. DMRT1 dosage probably increases progressively during human germline development, reaching higher levels during the mitotic arrest of PGCs (Fig. 1a)^24^. Regulation of DMRT1 levels might be crucial for the stage-specific role in the human foetal germline. A dose-sensitive regulation by DMRT1 has been reported in another context^59,82^.

DMRT1 repressed pluripotency factors, a hallmark of germline commitment towards mitotic arrest in male PGCs and pre-meiotic PGCs in female^24^ (Fig. 8b); suppression of DMRT1 in second-trimester human foetal testis induces upregulation of POU5F1 (ref. ^83^), and Dmrt1 depletion in 129Sv male mice causes germ cell teratoma through the lack of suppression of pluripotency genes^59,84^. Notably, genomic variants were identified near the DMRT1 locus in testicular germ cell tumours, indicating the potential origin of carcinoma in situ, and the role of DMRT1 for irreversible germline commitment^58,85–87^.

Our in vitro gene induction system did not allow an accurate temporal and dosage control of DMRT1 as is likely in vivo. Accordingly, DZ^+^PGCLCs exhibit enrichment of 5hmC with incomplete global DNA demethylation equivalent to the epigenetic status of migratory PGCs; instead, there was induction of the mitotic arrest transcriptional programme. A gradual increase in DMRT1 in vivo occurs over a few weeks leading step-wise towards mitotic arrest PGCs^24^. The high levels of DMRT1 in our system may trigger mitotic arrest more rapidly within 4–8 days, which potentially prevents DNA replication coupled dilution of 5mC in PGCLCs^88,89^. We also did not observe transcriptional repression of H3K9 methyltransferases, G9A and SETDB1 in DZ^+^PGCLCs, which accompany global DNA demethylation in PGCs in vivo^10,24^. Future studies with exquisitely tunable expression of DMRT1 are warranted to mimic the stage-specific role. Note that the lack DDX4 expression in DZ^+^PGCLCs as in in vivo mitotic arrest PGCs may occur if combined with gonadal somatic tissues (Extended Data Fig. 3j).

While our mechanistic studies on DMRT1 were performed using male cells, female DZ^+^PGCLCs also showed downregulation of pluripotency genes, *POU5F1* and *NANOG* (Extended Data Fig. 3i), indicating the RA-responsive phase in female. Expression of RA-responsive genes occurs in male DZ^+^PGCLCs and mitotic arrest PGCs^24^. DMRT1 declines in female PGCs with meiotic entry, following the RA-responsive stage^24^. Accordingly, there is probably a common role for DMRT1 for the germline commitment in males and females; DMRT1 downregulation may lead to meiosis in the female germline, but further studies with respect to female specific events, such as the status of the X chromosome, merit consideration in the future.

Our study implicates DMRT1 as an essential factor regulating the transition from nascent PGCs to gametogenesis-competent cells, involving locus-specific epigenetic resetting. We provide a critical foundation for further investigations and experimental approaches for advances in human germline biology and in vitro gametogenesis.

## Methods

### Ethics statement

Human embryonic tissues were used with permission from the National Health Service Research Ethical Committee, UK (Research Ethics Committee number 96/085). Patients (who had already decided to undergo the termination of pregnancy operation) fully and freely consented to donate the foetal tissues for medical and academic research. We received genital ridges and dissected to isolate gonads from mesonephric tissues. The gonadal tissues were dissociated into single-cell suspension with Collagenase IV (2.6 mg ml^−1^) (Sigma, C5138) and DNase I (10 U ml^−1^) in Dulbecco’s modified Eagle medium (DMEM)–F/12 (Gibco). Cells were resuspended in fluorescence-activated cell sorting (FACS) medium (phosphate-buffered saline (PBS) with 3% foetal calf serum) with 5 μl of Alexa Fluor 488 anti-alkaline phosphatase (BD Pharmingen, 561495) and 5 μl of PerCP–Cy5.5 anti-CDH5 (BD Pharmingen, 561566) antibodies for flow cytometry. Medical or surgical termination of pregnancy was carried out at Addenbrooke’s Hospital, Cambridge, UK. This study did not involve the use of human gametes, pre-implantation embryos or experimental models mimicking early human development. Where applicable, our study is compliant with the International Society for Stem Cell Research guidelines. All samples were handled and stored according to the Human Tissue Act regulations. The Gurdon Institute safety committee carried out appropriate scrutiny, including risk assessments.

### Collection of PGCs from human embryos

Crown–rump length and anatomical features, including limb and digit development, were used to determine the developmental stage of human embryos with reference to Carnegie staging. The sex of embryos was determined by sex determination PCR as previously described^90^. Genital ridges were dissected and separated from surrounding mesonephric tissues and dissociated into single-cell suspension with Collagenase IV (2.6 mg ml^−1^) (Sigma, C5138) and DNase I (10 U ml^−1^) in DMEM–F/12 (Gibco) at 37 °C for 15–30 min. Cells were resuspended in FACS medium (PBS with 3% foetal calf serum) with 5 μl of Alexa Fluor 488 anti-alkaline phosphatase (BD Pharmingen, 561495) and 5 μl of PerCP–Cy5.5 anti-CDH5 (BD Pharmingen, 561566) antibodies for 20 min at room temperature. Flow cytometry was performed with BD LSRFortessa Cell Analyzer (BD Biosciences), and dot plots were generated by FlowJo software.

### Cell culture

Approval for the use of all ES cell lines used in this study was granted by the MRC Steering Committee for the UK Stem Cell Bank and for the Use of Stem Cell Lines. Male ES cell line, WIS2 (46XY), was kindly provided by Weizmann Institute of Science, Israel^91^. Female ES cell line, Shef-6 (46XX), was obtained from the UK Stem Cell Bank (UKSCB accession no. R-05-031). 4i ES cells were maintained on irradiated mouse embryonic fibroblasts (MEFs) (purchased from MTI-GlobalStem or prepared in house) in knockout DMEM (Thermo Fisher Scientific) supplemented with 20% knockout serum replacement, 0.1 mM non-essential amino acids, 0.1 mM 2-mercaptoethanol, 100 U ml^−1^ penicillin, 0.1 mg ml^−1^ streptomycin, 2 mM l-glutamine, 20 ng ml^−1^ human LIF (Stem Cell Institute, University of Cambridge (SCI)), 8 ng ml^−1^ bFGF (SCI), 1 ng ml^−1^ TGFβ (Peprotech), 3 µM GSK3i (CHIR99021, Miltenyi Biotec), 1 µM ERKi (PD0325901, Miltenyi Biotec), 5 µM p38i (SB203580, TOCRIS Bioscience) and 5 µM JNKi (SP600125, TOCRIS Bioscience), as reported^14^. Cells were passaged every 2–4 days using TrypLE Express (Thermo Fisher Scientific). Before seeding 4i ES cells on MEFs, 10 µM of ROCKi (Y-27632, TOCRIS Bioscience) was added into the medium. Conventional ES cells were maintained on vitronectin (Thermo Fisher Scientific)-coated plates in Essential 8 medium (Thermo Fisher Scientific) according to the manufacturer’s protocol. Cells were passaged every 3–5 days using 0.5 mM ethylenediaminetetraacetic acid (EDTA)/PBS.

To induce PGCLCs, 4i ES cells or preME (see below) cells were trypsinized into single cells and seeded into Corning Costar Ultra-Low attachment multiwell 96-well plates (Sigma) or AggreWell Microwell Plates (Stemcell Technologies) at 4,000–8,000 cells per well. PGCLC induction medium based on aRB medium contains 500 ng ml^−1^ BMP2 (SCI), 100 ng ml^−1^ SCF (Peprotech), 50 ng ml^−1^ EGF (R&D Systems) and 10 µM ROCKi. aRB medium is composed of Advanced RPMI 1640 Medium (Thermo Fisher Scientific) supplemented with 1% B27 supplement (Thermo Fisher Scientific), 0.1 mM non-essential amino acids, 100 U ml^−1^ penicillin–0.1 mg ml^−1^ streptomycin and 2 mM l-glutamine^13^. For DM^+^PGCLC induction, PGCLC induction medium was replaced with aRB medium containing 100 ng ml^−1^ ActA (SCI), 20 µM Ra (Sigma), 100 ng ml^−1^ SCF (Peprotech) and 50 ng ml^−1^ EGF (R&D Systems) as indicated. For preME induction, trypsinized ES cells cultured in E8 were seeded on vitronectin-coated dish at 200,000 cells per well in 12-well plates in preME induction medium that is composed of aRB medium supplemented with 100 ng ml^−1^ ActA (SCI), 3 µM GSK3i and 10 µM ROCKi. For induction of exogenous transgenes, 100 µM DEX (Sigma) and/or 1 µg ml^−1^ dox (Sigma) was added.

### Vector construction and transfection

For construction of reporter knock-in targeting vector, 5′ and 3′ arms amplified from human genomic DNA and combined with tdTomato or mVenus and Rox–PGK–PuroΔtk–Rox were cloned into modified NANOS3–tdTomato targeting vector containing MC1-promoter-driven diphtheria toxin A using in-fusion HD cloning kit (Takara Bio)^13^. Guide RNAs targeting around the stop codon sequence of DMRT1 or DAZL genes (Supplementary Table 1) were cloned into pX330 (Addgene). For construction of dox-inducible system, DMRT1 and BCL2L1 were cloned into PiggyBAC pCMV–Tet3G vector used previously^13^. All fragments were amplified by PCR using PrimeSTAR MAX, PrimeSTAR GXL DNA polymerase (Takara Bio) or Q5 High-Fidelity DNA Polymerase (NEB) according to the manufacturer’s protocol.

Plasmid transfection for gene targeting or transgene introduction was carried out with electroporation or lipofection as described before^13,14^. In brief, electroporation was carried out using Gene Pulser equipment (Bio-Rad) with 1–5 × 10^6^ 4i ES cells mixed with targeting vector and pX330 plasmid containing guide RNA. For lipofection, reverse transfection was carried out with 2 × 10^5^ 4i ES cells in 100–200 µl of Opti-MEM containing plasmid vectors and Lipofectamine 2000 or Lipofectamine Stem Transfection Reagent (Thermo Fisher) with 5 min incubation at room temperature. After electroporation or lipofection, ES cells were seeded onto 4 drug resistant (DR4) MEFs (GlobalStem or SCI) and 48 h later, 0.5 µg ml^−1^ puromycin (Sigma) or 25 µg ml^−1^ hygromycin B (Thermo Fisher Scientific) was added to the culture medium for selection. Drug-resistant ES cell colonies were picked up and genotyped for correct targeting by PCR using primers in Supplementary Table 1. The targeted clones were expanded and then used for excision of Rox-flanked PGK–PuroΔtk by transient transfection of pCAG–Dre–IH. After selection with 25 µg ml^−1^ hygromycin B and subsequently with 0.2 µM fialuridine, colonies were picked up and assessed for excision by PCR using primers in Supplementary Table 1 (Extended Data Fig. 1c).

### qPCR

Total RNA was extracted using PicoPure RNA Isolation Kit (Thermo Fisher) and cDNA was synthesized using QuantiTect Reverse Transcription Kit (QIAGEN) according to manufacturer’s protocols. RT–qPCR was performed using QuantStudio 6 Flex Real-Time PCR System (Thermo Fisher). Primer sequences are listed in Supplementary Table 3. Values shown were normalized to housekeeping genes and relative changes to control sample values.

Genomic DNA was extracted using Quick-DNA Microprep Plus Kit (Zymo). Primer sequences for genomic DNA quantification are listed in Supplementary Table 4. Values shown were normalized to human genomic locus for TPOX and normalized to wild-type sample copy numbers.

### Immunofluorescence and image analysis

Aggregates were fixed in 4% paraformaldehyde for 1–2 h at 4 °C and embedded in OCT compound (VWR) for frozen sections. Sections were incubated with primary antibodies for 1–2 h at room temperature or overnight at 4 °C and with fluorescent-conjugated secondary antibodies (dilution 1:500) for 1 h at room temperature. Primary antibodies are listed in Supplementary Table 5 (anti-DMRT1, rabbit, monoclonal, Abcam, cat. no. ab166893, dilution 1:500; anti-POU5F1, mouse, monoclonal, BD Biosciences, cat. no. 611203, dilution 1:500; anti-DAZL, rabbit, polyclonal, Abcam, cat. no. ab34139, dilution 1:200; anti-5mC, rabbit, monoclonal, Cell Signaling Technology, cat. no. 28692, dilution 1:200; anti-5mC, mouse, monoclonal, Abcam, cat. no. ab10805, dilution 1:150; anti-5hmC, rabbit, polyclonal, active motif, cat. no. 39769, dilution 1:500; anti-DNMT3B, sheep, polyclonal, R&D Systems, cat. no. AF7646, dilution 1:200; anti-TFAP2C, rabbit, polyclonal, Santa Cruz Biotechnology, cat. no. sc-8977, dilution 1:200; anti-SOX9, goat, polyclonal, R&D Systems, cat. no. AF3075-SP, dilution 1:200; anti-tdTomato, goat, polyclonal, SICGEN, cat. no. AB8181, dilution 1:100; anti-DDX4, rabbit, monoclonal, Abcam, cat. no. 235442, dilution 1:200; anti-mitochondria, mouse, monoclonal, Abcam, cat. no. ab92824, dilution 1:800; anti-SOX17, goat, polyclonal, R&D Systems, cat. no. AF1924, dilution 1:100; APC conjugated SUSD2, mouse, monoclonal, BioLegend, cat. no. 327408, dilution 1:100; anti-TFCP2L1, goat, polyclonal, R&D Systems, cat. no. AF5726, dilution 1:100). After antibody treatment, sections were stained with 4′,6-diamidino-2-phenylindole (Sigma) and imaged using Leica SP8 inverted laser scanning confocal microscope by white laser. HC PL APO CS2 63× 1.4 numerical aperture oil immersion objective was used. Image analyses were performed using a custom script^92^ for Fiji^93^, which segments nuclei in 4′,6-diamidino-2-phenylindole channel with difference of Gaussian threshold using Otsu’s method^94^ and measures intensity in channels for 5mC, 5hmC.

### Flow cytometry analysis

Aggregates were trypsinized with trypsin/EDTA (0.25%, Thermo Fisher) at 37 °C for 5–15 min and single-cell suspension was incubated with Alexa Fluor 488 or 647 conjugated anti-alkaline phosphatase (TNAP) antibody (BD Bioscience, 5 µl per sample), PerCP–Cy5.5-conjugated anti-CDH5 antibody (BioLegend, 5 µl per sample) and/or Alexa Fluor 647 conjugated anti-CD38 antibody (BioLegend, 5 µl per sample) and analysed using BD LSRFortessa Cell Analyzer (BD Bioscience). Flow cytometry data were analysed using FlowJo software.

### Luciferase assay

For vector construction of luciferase assay, three genomic regions with DMRT1 binding peaks containing DMRT1 motif (hg38; peak 1: chr3:16,608,590–16,608,949, DMRT1 motif: aaaactatgttact; peak 2: chr3:16,602,880–16,603,116, DMRT1 motif: aatacatagtagta; peak 3: chr3:16,594,400–16,597,625 DMRT1 motif: ttgatacaatgttt) in day 4 DZ^+^PGCLCs at DAZL locus were amplified from human genomic DNA. These sequences were cloned into a piggyBAC-based luciferase (Luc+) reporter plasmid containing a hygromycin-resistant gene driven by a PGK promoter using in-fusion HD cloning kit. DMRT1 motif is scanned using HOMER scanMotifGenomeWide.pl function. The sequences without DMRT1 motif were amplified from the original plasmid with each peak’s sequences using the primers listed in Supplementary Table 2. ALR/alpha consensus sequences (aattctcagtaacttccttgtgttgtgtgtattcaactcacagagttgaacgatcctttacacagagcagacttgaaacactctttttgtggaatttgcaagtggagatttcagccgctttgaggtcaatggtagaataggaaatatcttcctatagaaactagacagaat, DMRT1 motif sequence: ttgaaacactctttt) were downloaded from Repbase. The synthesized ALR oligos from Merck were cloned into a piggyBAC-based luciferase (Luc+) reporter plasmid containing a hygromycin-resistant gene driven by a PGK promoter using in-fusion HD cloning kit.

HEK 293 cells (ATCC CRL-1573) were transfected using Lipofectamine 2000 Transfection Reagent (Thermo Fisher) with a piggyBAC plasmid containing a constitutively expressed green fluorescent protein (GFP) cassette and a neomycin-resistant cassette, a piggyBAC plasmid containing a dox-inducible DMRT1 transgene and a puromycin-resistant cassette, and a plasmid encoding a piggyBAC transposase. Following 4 days of ±dox treatment, cells were measured for GFP with Hidex Sense (HIDEX) and subjected to luciferase activity assay using the Dual-Glo Luciferase Assay System (Promega). Normalized luciferase activities were obtained by dividing firefly luciferase activity by GFP signal counts.

### Western blot

Nuclear proteins were extracted using EpiQuik Nuclear Extraction Kit II (EPIGENTEK) and were separated on a Novex 4–20% Tris-Glycine Mini Gel (Thermo Fisher) using XCell SureLock Mini-Cell Electrophoresis System (Thermo Fisher) and transferred to Hybond P 0.45 µm polyvinylidene fluoride membrane (GE Healthcare). After blocking in 5% skimmed milk, the membrane was incubated with primary antibodies (anti-SOX17, rabbit, monoclonal, Cell Signaling Technology, cat. no. 81778, dilution 1:1,000; anti-PRDM1, rabbit, monoclonal, Cell Signaling Technology, cat. no. 9115, dilution 1:500; anti-DMRT1, rabbit, monoclonal, Abcam, cat. no. ab126741, dilution 1:1,000; anti-LaminB1, rabbit, polyclonal, Abcam, cat. no. ab16048, dilution 1:1,000; Supplementary Table 6). The antibody binding was detected by horseradish-peroxidase-conjugated anti-rabbit IgG (Dako; dilution 1:2,000 in 0.01% TBST) in conjunction with the Western Detection System (GE Healthcare).

### Preparation of scRNA-seq libraries

Reporter or cell surface marker-positive cells were sorted by BD FACSAria III Cell Sorter and loaded according to the manufacturer’s protocol for the Chromium Next GEM Single Cell 3′ Reagent Kits v3.1 (Dual Index) (10x Genomics) to attain between 2,000 and 6,000 cells per reaction. Library preparation was carried out according to the manufacturer’s protocol. Libraries were sequenced, aiming at a minimum coverage of 40,000 raw reads per cell, on the Novaseq 6000 systems using the sequencing format: read 1, 28 cycles; i7 index, 10 cycles; i5 index, 10 cycles; read 2, 90 cycles.

### Preparation of bulk RNA-seq libraries

RNA-seq library was generated with total RNA (300 ng) using NEBNext Ultra II Directional RNA Library Prep Kit for Illumina (E7760, NEB) with NEBNext rRNA Depletion Kit v2 (NEB) according to manufacturer’s protocol. Library was quantified using NEBNext Library Quant Kit Quick Protocol (E7630, NEB). Libraries were sequenced for 150 cycles in paired-end mode on the NovaSeq platform.

### C&R

C&R for DMRT1 and normal rabbit IgG was performed as described^43–45^. Briefly, 50,000 purified DZ^+^PGCLCs were washed and bound to activated 10 μl Concanavalin A-coated magnetic beads. The beads were then incubated with wash buffer (20 mM HEPES, pH 7.5, 150 mM NaCl, 0.5 mM spermidine and protease inhibitor) containing 0.1% digitonin and 1 μg of DMRT1 antibody (ab126741, Abcam) or normal rabbit IgG (#2729, Cell signaling) for 2 h at 4 °C on a rotator. After two washes in digitonin–wash buffer, beads were resuspended in Protein A/G-MNase fusion protein at 70 ng ml^−1^ in digitonin–wash buffer and incubated for 1 h at 4 °C on a rotator. After two washes in digitonin–wash buffer (the beads with replicate 3 of day 4 DZ^+^PGCLC and day 8 DZ^+^PGCLC were washed with low-salt rinse buffer (20 mM HEPES, pH 7.5, 0.5 mM spermidine and 0.1% digitonin) once additionally), beads were resuspended in ice-cold calcium incubation buffer (3.5 mM HEPES pH 7.5, 10 mM CaCl_2_ and 0.1% digitonin). After 15 min, 2× stop buffer (340 mM NaCl, 20 mM EDTA, 4 mM egtazic acid, 0.1% digitonin, RNase A 100 μl ml^−1^ and glycogen 50 μg ml^−1^) was added. Beads were incubated at 37 °C for 30 min, the liquid was removed to a fresh tube and DNA was extracted with phenol–chloroform extraction.

### DNA library preparation and sequencing

Sequencing libraries were prepared with the NEBNext Ultra II DNA Library Prep Kit (NEB, E7645S) for Illumina according to the manufacturer’s protocol but without size selection and PCR enrichment of adaptor-ligated DNA. PCR enrichment of adaptor-ligated DNA was performed with KAPA HiFi Real-Time PCR Library Amplification Kit (Roche, KK2702) following the manufacturer’s recommendations. The number of PCR cycles using the KAPA polymerase was 7–10. SPRIselect beads (Beckman Coulter, B23317) were used for clean-up PCR product and size selection. Libraries were sequenced for 150 cycles in paired-end mode on the NovaSeq platform.

### TAPS with βGT blocking and chemical-assisted pyridine borane sequencing plus

TAPS with βGT blocking (TAPSβ) and chemical-assisted pyridine borane sequencing plus (CAPS+) were performed according to previous publications^47,48^. Briefly, DNA was spiked with spike-in control DNA and sonicated to 300–500 bp, before ligation with NEBNext Adaptor for Illumina using KAPA HyperPrep Kit according to the manufacturer’s protocol. The uracil in the loop of NEBNext Adaptor was removed by USER Enzyme (New England Biolabs). A total of 100 ng ligated DNA was used for both TAPSβ and CAPS+. For TAPSβ, the ligated library was subjected to βGT (Thermo Fisher) blocking, two rounds of mTet1 oxidation, and borane reduction. For CAPS+, the ligated library was subjected to chemical oxidation and borane reduction. Converted DNA from TAPSβ and CAPS+ was amplified with NEBNext Multiplex Oligos for Illumina and KAPA HiFi HotStart Uracil+ ReadyMix PCR Kit for four cycles according to the manufacturer’s protocol. The PCR product was purified with Ampure XP beads. Libraries were sequenced for 150 cycles in paired-end mode on the NovaSeq 6000 platform.

### Data processing for scRNA-seq

The reads were demultiplexed and aligned to the 10x Genomics’ GRCh38-2020-A reference genomes using the Cell Ranger Software (v.7.0.0, 10x Genomics) with default parameters. The summary statistics from Cell Ranger is provided in Supplementary Table 7.

We employed Scrublet to identify and distinguish single cells from cell doublets in each individual library. As described in ref. ^95^, we used a two-step diffusion doublet identification followed by Bonferroni–false discovery rate (FDR) correction and a significance threshold of 0.01. We used Scanpy v.1.8.0 (ref. ^96^) to analyse the filtered count matrices that were generated by Cell Ranger, following their recommended standard practices. Specifically, we excluded genes that were expressed by fewer than three cells and excluded cells that expressed fewer than 3,000 genes or had more than 10% mitochondrial content. We then normalized the raw counts by library size and log-transformed them. Next, we identified the highly variable genes, which we used for principal components analysis (PCA). We corrected for the library effect using Harmony^97^ on the PCA space (default parameters except theta = 1). Finally, we used the Harmony-corrected PCA space to identify the *k* (*k* = 15) nearest neighbours, perform Leiden clustering and visualize the results using UMAP. Leiden clusters with overall high doublet score or low counts number were flagged and discarded in further analysis. We used Seurat’s v.4.0.5 FindAllMarkers() function to identify up- and downregulated genes in each library with |log_2_fold change (FC)| >1 (ref. ^98^). To determine the cell cycle phase (that is, G1, S or G2/M) of each cell, we combined the expression of G2/M and S phase markers and used the method implemented in Scanpy’s score_genes_cell_cycle function to classify the cells^99^. We then compared the in vitro cell states identified in our study with the in vivo cell states reported in the Smart-seq2 dataset of gonadal cells from Li et al.^24^ (GSE86146). To do this, we downloaded the normalized transcripts per million (TPM) matrix from Li et al.^24^ and annotated their cells using the ‘FullAnnot’ field. We only considered the male foetal germ cell clusters. We used the tool scmap^100^ to project the Li et al.^24^ annotations onto our dataset and visualized the results of the projections using a dot plot.

### Data processing for bulk RNA-seq

Trim Galore^101^ was used to remove the low-quality reads and adaptor sequences. Trimmed sequence files were mapped to human reference genome (GENCODE, GRCh38.p13) and counts on genes were generated using STAR^102^ with parameters –outFilterMultimapNmax 1 –outFilterMatchNmin 35. Normalized counts (normalize the total number of mapped reads per experiment to 1 × 10^8^) on repeat elements were generated with the analyzeRepeats.pl of the HOMER^103^ package. Differential gene (or repeat element) expression analysis was performed with the glm method of the edgeR^104^ package for protein-coding genes. DEGs or repeat elements were identified with fold changes greater than 2 and FDR smaller than 0.05. Reads per kilobase of transcript per million mapped reads (RPKM) values of genes were calculated using Cufflinks^105^.

Secondary data analyses were performed using Microsoft Excel and R software version 4.0.5 with the packages ggplot2. GSEA^106^ was performed using the GSEA software by the Broad Institute. GO analysis was performed on the basis of GO Biological Process (http://geneontology.org). Marker protein-coding genes, 142 for migratory, 288 for mitotic and 937 for mitotic arrest male PGCs, were used on the basis of published markers identified from single cell RNA-seq data^24,28^, ‘PGC genes’ were identified on the basis of shared enriched DEGs (logFC >1, FDR <0.05) between week 7 and week 9 male PGCs against week 7 gonadal somatic cells or conventional ES cells^6^.

### Data processing for C&R

To trim the short fragments that are frequently encountered in C&R experiments we used leeHom package program^107^ with —ancientdna option. The trimmed reads were aligned to the human reference genome (GENCODE, GRCh38.p13) using Bowtie2 2.2.6 (ref. ^108^) with options –very-sensitive –no-mixed –no-discordant -q –phred33 -I 10 -X 700. For MACS2 peak calling, parameters used were macs2 (ref. ^109^) callpeak –keep-dup all and the peaks with −log_10_(*q* value) >10 for day 4 DZ^+^PGCLC and the peaks with −log_10_(*q* value) >8 with IgG as control for day 8 DZ^+^PGCLC were selected. A total of 11,920 (day 4) and 7,818 (day 8) peaks that are in common between the replicates were used for further analysis. Peaks were annotated to their nearest genes or overlapping repeat elements using Homer annotatePeaks.pl function. To analyse the enriched TF motifs over peaks or repeat elements, HOMER findMotifsGenome.pl function was used.

### Data processing for TAPSβ and CAPS+ methylome

The reads were demultiplexed using i7 sequences. The total sequencing reads number and conversion rate are provided in Supplementary Table 8. Trim Galore was used to remove the low-quality reads, and Samtools rmdup function was used to remove PCR duplicates. Trimmed reads were mapped to human reference genome (GENCODE, GRCh38.p13), and modified bases were called by asTair^110^. The methylation rate (%) for each CpG was calculated as the ratio between T and (C + T). Average CpG methylation levels of annotated genomic regions were calculated using UCSC bigWigAverageOverBed considering only information from CpGs with >5× coverage. To identify DMRs, we used DMRfinder^111^ with the default setting except –meanDiff_cutoff (5mC, 0.2; 5hmC, 0.05) and –pctMinCtrl 0 –pctMinExp 0 as sets of CpGs with a *t*-statistic greater than the critical value for *α* = 0.05 and with a gap <300 bases.

### Statistics and reproducibility

For RNA-seq, C&R and 5hmC/5mC methylome data, two independent biological replicates (except for day 4 DZ^+^PGCLC C&R with three independent biological replicates) were included according to the guidelines of the Encode Consortium101. No statistical method was used to pre-determine sample size in other experiments. Low-quality replicates of libraries were excluded from the analysis, as determined by percentage of reads in peaks, number of peaks and genome browser visualization. As all results involved equipment-based quantitative measure and no subjective rating of data was involved, blinding and randomization are not relevant. All the data met the assumptions of the statistical tests used, including whether normality and equal variances were formally tested. All the data collection and analysis were not performed blind to the conditions of the experiments.

### Reporting summary

Further information on research design is available in the Nature Portfolio Reporting Summary linked to this article.

## Online content

Any methods, additional references, Nature Portfolio reporting summaries, source data, extended data, supplementary information, acknowledgements, peer review information; details of author contributions and competing interests; and statements of data and code availability are available at 10.1038/s41556-023-01224-7.

### Supplementary information

Reporting Summary

Supplementary Tables 1–8 (1) Oligos. (2) DNA cloning primers for luciferase assay. (3) Primers for RT–qPCR. (4) Primers for genomic DNA qPCR. (5) Antibodies for immunofluorescence. (6) Antibodies for western blot. (7) Summary statistics from Cell Ranger for scRNA-seq. (8) Total reads number and conversion rate for CAPS+ and TAPSβ whole-genome sequencing.

### Source data

Source Data Fig. 1 Statistical source data.

Source Data Fig. 2 Statistical source data.

Source Data Fig. 3 Statistical source data.

Source Data Fig. 6 Statistical source data.

Source Data Fig. 7 Statistical source data.

Source Data Extended Data Fig. 1 Unprocessed western blots and agarose gel images.

Source Data Extended Data Table 1 Statistical source data.

Source Data Extended Data Fig. 3 Statistical source data.

Source Data Extended Data Fig. 3 Unprocessed western blots and agarose gel images.

## Data Availability

Sequencing data that support the findings of this study have been deposited in the Gene Expression Omnibus (GEO) under accession code GSE223036. Source data are provided with this paper. All other data supporting the findings of this study are available from the corresponding authors on reasonable request.

## Code availability

The script we used for the image analysis is released under the GPL v3, which is included in the GitHub repository (https://github.com/gurdon-institute/Nucleus_Measure/blob/main/LICENSE) and allows anyone to use, modify and distribute the software under the same license without warranty or liability.
