# Supplementary material for: DMRT1 regulates human germline commitment
Source: Nat Cell Biol. 2023 Sep 14;25(10):1439–52. doi: 10.1038/s41556-023-01224-7 (PMC10567552; doi:10.1038/s41556-023-01224-7)
Supplement: Supplementary file 1 — Reporting Summary [file 41556_2023_1224_MOESM1_ESM.pdf]

nature portfolio

Corresponding author(s): Naoko Irie, M. Azim Surani

Last updated by author(s): 17 July 2023

Reporting Summary

Nature Portfolio wishes to improve the reproducibility of the work that we publish. This form provides structure for consistency and transparency in reporting. For further information on Nature Portfolio policies, see our Editorial Policies and the Editorial Policy Checklist.

Statistics

For all statistical analyses, confirm that the following items are present in the figure legend, table legend, main text, or Methods section.

n/a	Confirmed	
☐	☒	The exact sample size (*n*) for each experimental group/condition, given as a discrete number and unit of measurement
☐	☒	A statement on whether measurements were taken from distinct samples or whether the same sample was measured repeatedly
☐	☒	The statistical test(s) used AND whether they are one- or two-sided<br>*Only common tests should be described solely by name; describe more complex techniques in the Methods section.*
☐	☒	A description of all covariates tested
☐	☒	A description of any assumptions or corrections, such as tests of normality and adjustment for multiple comparisons
☐	☒	A full description of the statistical parameters including central tendency (e.g. means) or other basic estimates (e.g. regression coefficient) AND variation (e.g. standard deviation) or associated estimates of uncertainty (e.g. confidence intervals)
☐	☒	For null hypothesis testing, the test statistic (e.g. *F*, *t*, *r*) with confidence intervals, effect sizes, degrees of freedom and *P* value noted<br>*Give P values as exact values whenever suitable.*
☒	☐	For Bayesian analysis, information on the choice of priors and Markov chain Monte Carlo settings
☒	☐	For hierarchical and complex designs, identification of the appropriate level for tests and full reporting of outcomes
☒	☐	Estimates of effect sizes (e.g. Cohen's *d*, Pearson's *r*), indicating how they were calculated

Our web collection on statistics for biologists contains articles on many of the points above.

Software and code

Policy information about availability of computer code

Data collection	-FACS was performed using BD LSRFortessa Cell Analyzer (BD Bioscience)<br>-qPCR was performed on a QuantStudio 6 Flex Real-Time PCR System (Thermo Fisher)<br>-Immunofluorescences were performed using Leica SP8 inverted laser scanning confocal microscope<br>-All libraries were sequenced using NovaSeq 6000 (Illumina)
Data analysis	-Flow cytometry data was analysed using FlowJo (10.7.1)<br>-Immunofluorescences data was analysed using Fiji software (2.9.0) and a custom script (https://github.com/gurdon-institute/Nucleus_Measure/blob/main/Nucleus_Measure.py)<br>-For quantification in qPCR analysis, The ΔCt or ΔΔCt method was used.<br>-Bioinformatic software: Trim Galore (0.6.6), STAR (2.5.4b), cufflinks (2.2.1), R (4.0.5), edgeR (3.36.0), leeHom (1.2.5), asTair (3.3.2), cutadapt (1.15), Bowtie 2 (2.2.6), samtools (1.7), MACS2 (2.1.2), HOMER (4.11.1), bedtools (2.26.0), Seurat (v4.0.5), Cell Ranger (7.0.0), Scanpy (1.8.0), IGV (2.15.1), GSEA (4.1.0)

For manuscripts utilizing custom algorithms or software that are central to the research but not yet described in published literature, software must be made available to editors and reviewers. We strongly encourage code deposition in a community repository (e.g. GitHub). See the Nature Portfolio guidelines for submitting code & software for further information.

Data

Policy information about availability of data

All manuscripts must include a data availability statement. This statement should provide the following information, where applicable:

- Accession codes, unique identifiers, or web links for publicly available datasets
- A description of any restrictions on data availability
- For clinical datasets or third party data, please ensure that the statement adheres to our policy

Bulk RNA sequencing datasets, single cell sequencing datasets, methylome and CUT&RUN sequencing data sets have been deposited in NCBI GEO with the accession code GSE223036.
Genome databases used are: Gencode Human Release GRC38.p13 and 10X Genomics GRCh38-2020-A. Source data are provided with this study. All other data supporting the findings of this study are available from the corresponding author on reasonable request.

Field-specific reporting

Please select the one below that is the best fit for your research. If you are not sure, read the appropriate sections before making your selection.

☒ Life sciences ☐ Behavioural & social sciences ☐ Ecological, evolutionary & environmental sciences

For a reference copy of the document with all sections, see nature.com/documents/nr-reporting-summary-flat.pdf

Life sciences study design

All studies must disclose on these points even when the disclosure is negative.

Sample size	For NGS libraries, two or three biological replicates were generated according to the practice of the Encode Consortium. For other experiments, no sample size calculation was performed. As per standard practice in molecular and cell biology, at least 2 replicates were generated.
Data exclusions	Low quality replicate of CUT&RUN libraies were excluded from the analysis.
Replication	The experimental findings were reliably reproduced with at least two or three replications.
Randomization	In each experiment, cells started from the same conditions and treatments were randomly allocated to experimental groups.
Blinding	All results involved equipment-based quantitative measure and no subjective rating of data was involved, hence blinding is not relevant.

Reporting for specific materials, systems and methods

We require information from authors about some types of materials, experimental systems and methods used in many studies. Here, indicate whether each material, system or method listed is relevant to your study. If you are not sure if a list item applies to your research, read the appropriate section before selecting a response.

Materials & experimental systems

n/a	Involved in the study	
☐	☒	Antibodies
☐	☒	Eukaryotic cell lines
☒	☐	Palaeontology and archaeology
☒	☐	Animals and other organisms
☐	☒	Human research participants
☒	☐	Clinical data
☒	☐	Dual use research of concern

Methods

n/a	Involved in the study	
☐	☒	ChIP-seq
☐	☒	Flow cytometry
☒	☐	MRI-based neuroimaging

Antibodies

Antibodies used	The primary antibodies used for immunofluorescence:<br>1. anti-DMRT1, Rabbit, Monoclonal, Abcam, Cat. number ab166893, Lot number GR119578-4 (1:500)<br>2. anti-POU5F1, Mouse, Monoclonal, BD Biosciences, Cat. number 611203, Lot number 8087969 (1:500)<br>3. anti-DAZL, Rabbit, Polyclonal, Abcam, Cat. number ab34139, Lot number GR3184650-3 (1:200)<br>4. anti-5mC, Rabbit, Monoclonal, Cell Signaling Technology, Cat. number 28692, Lot number 2 (1:200)<br>5. anti-5mC, Mouse, Monoclonal, Abcam, Cat. number ab10805, Lot number GR3390032-1 (1:150)<br>6. anti-5hmC, Rabbit, Polyclonal, Active Motif, Cat. number 39769, Lot number 21518003 (1:500)<br>7. anti-DNMT3B, Sheep, Polyclonal, R&D Systems, Cat. number AF7646, Lot number CGWF0118031 (1:200)

8. anti-TFAP2C, Rabbit, Polyclonal, Santa Cruz Biotechnology, Cat. number sc-8977, Lot number H0715 (1:200)
9. anti-SOX9, Goat, Polyclonal, R&D Systems, Cat. number AF3075-SP, Lot number WIL0418111 (1:200)
10. anti-tdTomato, Goat, Polyclonal, SICGEN, Cat. number AB8181, Lot number 81221018 (1:100)
11. anti-DDX4, Rabbit, Monoclonal, Abcam, Cat. number 235442, Lot number GR3233447-1 (1:200)
12. anti-Mitochondria, Mouse, Monoclonal, Abcam, Cat. number ab92824, Lot number GR3307132-3 (1:800)
13. anti-SOX17, Goat, Polyclonal, R&D Systems, Cat. number AF1924, Lot number KGA1019061 (1:100)
14. APC conjugated SUSD2, Mouse, Monoclonal, BioLegend, Cat. number 327408, Lot number B218305 (1:100)
15. anti-TFCP2L1, Goat, Polyclonal, R&D Systems, Cat. number AF5726, Lot number CCUG0219121 (1:100)

The antibodies used for FACS:
1. Alexa Fluor 647-conjugated anti-alkaline phosphatase, Mouse, Monoclonal, BD Pharmingen, Cat. number 561500, Lot number 7132684 (5 µl/ sample)
2. Alexa Fluor 488-conjugated anti-alkaline phosphatase, Mouse, Monoclonal, BD Pharmingen, Cat. number 561495, Lot number 7132712 (5 µl/ sample)
3. PerCP-Cy5.5 conjugated anti-CDH5, Mouse, Monoclonal, BD Pharmingen, Cat. number 561566, Lot number B316764 (5 µl/ sample)
4. Alexa Fluor 647 conjugated anti-CD38, Mouse, Monoclonal, BioLegend, Cat. number 303514, Lot number B170786 (5 µl/ sample)

The antibodies used for WB:
1. anti-SOX17, Rabbit, Monoclonal, Cell Signaling Technology, Cat. number 81778, Lot number 1 (1:1000)
2. anti-PRDM1, Rabbit, Monoclonal, Cell Signaling Technology, Cat. number 9115, Lot number 6 (1:500)
3. anti-DMRT1, Rabbit, Monoclonal, Abcam, Cat. number ab126741, Lot number YI081707CS (1:1000)
4. anti-LaminB1, Rabbit, Polyclonal, Abcam , Cat. number ab16048, Lot number GR3244626-1 (1:1000)
5. horseradish-peroxidase-conjugated anti-rabbit IgG, Goat, Polyclonal, Agilent, Cat. number P0448, Lot number 20023997 (1:2000)

Validation

No home made antibodies were used in this study. All IF, WB and FACS antibodies were commercially validated as below:

anti-DMRT1, Abcam, Cat. number ab166893, Lot number GR119578-4, Clone number, EPR6936-209
Validation: https://www.abcam.com/products/primary-antibodies/dmrt1-antibody-epr6936-209-ab166893.html

anti-POU5F1, BD Biosciences, Cat. number 611203, Lot number 8087969, Clone number, 40/Oct-3
Validation: https://www.bdbiosciences.com/en-gb/products/reagents/microscopy-imaging-reagents/immunofluorescence-reagents/purified-mouse-anti-oct3-4.611203

anti-DAZL, Abcam, Cat. number ab34139, Lot number GR3184650-3
Validation: https://www.abcam.com/products/primary-antibodies/dazl-antibody-ab34139.html

anti-5mC, Cell Signaling Technology, Cat. number 28692, Lot number 2, Clone number, D3S2Z
Validation: https://www.cellsignal.com/products/primary-antibodies/5-methylcytosine-5-mc-d3s2z-rabbit-mab/28692

anti-5mC, Abcam, Cat. number ab10805, Lot number GR3390032-1, Clone number, 33D3
Validation: https://www.abcam.com/products/primary-antibodies/5-methylcytosine-5-mc-antibody-33d3-ab10805.html"

anti-5hmC, Active Motif, Cat. number 39769, Lot number 21518003
Validation: https://www.activemotif.com/catalog/details/39769/5-hydroxymethylcytidine-5-hmc-antibody

anti-DNMT3B, R&D Systems, Cat. number AF7646, Lot number CGWF0118031
Validation: https://www.rndsystems.com/products/human-dnmt3b-antibody_af7646

anti-TFAP2C, Santa Cruz Biotechnology, Cat. number sc-8977, Lot number H0715
Validation: https://www.scbt.com/p/ap-2gamma-antibody-h-77?productCanUrl=ap-2gamma-antibody-h-77&_requestid=1379409

anti-SOX9, R&D Systems, Cat. number AF3075-SP, Lot number WIL0418111
Validation: https://www.rndsystems.com/products/human-sox9-antibody_af3075

anti-tdTomato, SICGEN, Cat. number AB8181, Lot number 81221018
Validation: https://www.labome.com/product/SICGEN/AB8181-200.html

anti-DDX4, Abcam, Cat. number 235442, Lot number GR3233447-1, Clone number, EPR21789
Validation: https://www.abcam.com/products?keywords=235442

anti-Mitochondria, Abcam, Cat. number ab92824, Lot number GR3307132-3, Clone number, 113-1
Validation: https://www.abcam.com/products/primary-antibodies/mitochondria-antibody-113-1-bsa-and-azide-free-ab92824.html

anti-SOX17, R&D Systems, Cat. number AF1924, Lot number KGA1019061
Validation: https://www.rndsystems.com/products/human-sox17-antibody_af1924

APC conjugated SUSD2, BioLegend, Cat. number 327408, Lot number B218305, Clone number, W5C5
Validation: https://www.biolegend.com/en-gb/products/apc-anti-human-susd2-antibody-8410

anti-TFCP2L1, R&D Systems, Cat. number AF5726, Lot number CCUG0219121
Validation: https://www.rndsystems.com/products/human-tfcp2l1-antibody_af5726

Alexa Fluor 647-conjugated anti-alkaline phosphatase, BD Pharmingen, Cat. number 561500, Lot number 7132684, Clone number, B4-78
Validation: https://www.bdbiosciences.com/en-us/products/reagents/flow-cytometry-reagents/research-reagents/single-color-antibodies-ruo/alexa-fluor-647-mouse-anti-human-alkaline-phosphatase.561500

Alexa Fluor 488-conjugated anti-alkaline phosphatase, BD Pharmingen, Cat. number 561495, Lot number 7132712, Clone number, B4-78
Validation: https://www.bdbiosciences.com/en-us/products/reagents/flow-cytometry-reagents/research-reagents/single-color-antibodies-ruo/alexa-fluor-488-mouse-anti-human-alkaline-phosphatase.561495

PerCP-Cy5.5 conjugated anti-CDH5, BD Pharmingen, Cat. number 561566, Lot number B316764, Clone number, 55-7H1
Validation: https://www.bdbiosciences.com/en-us/products/reagents/flow-cytometry-reagents/research-reagents/single-color-antibodies-ruo/percp-cy-5-5-mouse-anti-human-cd144.561566

Alexa Fluor 647 conjugated anti-CD38, BioLegend, Cat. number 303514, Lot number B170786, Clone number, HIT2
Validation: https://www.biolegend.com/en-gb/products/alexa-fluor-647-anti-human-cd38-antibody-3264

anti-SOX17, Cell Signaling Technology, Cat. number 81778, Lot number 1, Clone number, D1T8M
Validation: https://www.cellsignal.com/products/primary-antibodies/sox17-d1t8m-rabbit-mab/81778

anti-PRDM1, Cell Signaling Technology, Cat. number 9115, Lot number 6, Clone number, C14A4
Validation: https://www.cellsignal.com/products/primary-antibodies/blimp-1-prdi-bf1-c14a4-rabbit-mab/9115

anti-DMRT1, Abcam, Cat. number ab126741, Lot number YI081707CS, Clone number, EPR6936
Validation: https://www.abcam.com/products/primary-antibodies/dmrt1-antibody-epr6936-ab126741.html

anti-LaminB1, Abcam , Cat. number ab16048, Lot number GR3244626-1
Validation: https://www.abcam.com/products/primary-antibodies/lamin-b1-antibody-nuclear-envelope-marker-ab16048.html

horseradish-peroxidase-conjugated anti-rabbit IgG, Agilent, Cat. number P0448, Lot number 20023997
Validation: https://www.agilent.com/en/product/specific-proteins/elisa-kits-accessories/goat-anti-rabbit-immunoglobulins-hrp-affinity-isolated-2717113

Eukaryotic cell lines

Policy information about cell lines

Cell line source(s)

NANOS3-tdTomato and DMRT1-Venus reporter WIS2 line, NANOS3-Venus and DMRT1-tdTomato reporter WIS2 line, DAZL-tdTomato reporter WIS2 line, DMRT1-tdTomato reporter Shef-6 line, DAZL-tdTomato reporter Shef-6 line, NANOS3-tdTomato and DMRT1-Venus reporter WIS2 line bearing Dex-inducible SOX17/dox inducible PRDM1 transgenes, and DAZL-tdTomato reporter WIS2/Shef-6 lines bearing Dex-inducible SOX17/dox inducible DMRT1 transgenes were generated in this study according to the methods section.
HEK 293 cells (CRL-1573) were obtained from ATCC.

Authentication

Authentication of ESCs and PGCLCs by PCR, qPCR, RNAseq and immunofluorescence.

Mycoplasma contamination

All lines tested negative for mycoplasma contamination.

Commonly misidentified lines
(See ICLAC register)

No commonly misidentified lines were used for this study.

Human research participants

Policy information about studies involving human research participants

Population characteristics

Human embryonic genital ridges were collected from 2 individual embryos (wk7 Male and wk8 Female) for FACS analysis as shown in Figure 1b. Patient's identity is anonymised and authors have no access to donors' meta data.

Recruitment

Patients (who had already decided to undergo the termination of pregnancy operation) fully and freely consented to donate the foetal tissues for medical and academic research. Medical or surgical termination of pregnancy was carried out at Addenbrooke's Hospital, Cambridge, UK.

Ethics oversight

Human embryonic tissues were used under permission from National Health Service Research Ethical Committee, UK (REC Number: 96/085)

Note that full information on the approval of the study protocol must also be provided in the manuscript.

ChIP-seq

Data deposition

☒ Confirm that both raw and final processed data have been deposited in a public database such as GEO.

☒ Confirm that you have deposited or provided access to graph files (e.g. BED files) for the called peaks.

Data access links
May remain private before publication.

https://www.ncbi.nlm.nih.gov/geo/query/acc.cgi?&acc=GSE223036 using the reviewer token - grgruemubzsxfwp

Files in database submission	Fastq files of three DMRT1 CUT&RUN libraries. We provided the peaks information (BED) in Table 3.
Genome browser session (e.g. UCSC)	Bigwig files for the replicates are available in GSE223036. IGV Genome browser can be used for visualisation.

Methodology

Replicates	Three (d4 DZ+PGCLC), Two (d8 DZ+PGCLC)
Sequencing depth	2 × 150 bp paired-end run.<br><br>DMRT1 CUT&RUN in d4 DZ+PGCLC<br>Replicate 1 : 31912103 total number of reads, 17557224 uniquely mapped reads<br>Replicate 2 : 33388562 total number of reads, 19567846 uniquely mapped reads<br>Replicate 3 : 13604948 total number of reads, 12065492 uniquely mapped reads<br><br>DMRT1 CUT&RUN in d8 DZ+PGCLC<br>Replicate 1 : 56903866 total number of reads, 48185963 uniquely mapped reads<br>Replicate 2 : 51774414 total number of reads, 42507284 uniquely mapped reads<br><br>Normal Rabbit IgG CUT&RUN in d8 DZ+PGCLC<br>Replicate 1 : 43518396 total number of reads, 36337216 uniquely mapped reads
Antibodies	Rabbit monoclonal [EPR6936] to DMRT1 (Abcam, ab126741), Normal Rabbit IgG (#2729, Cell signaling)
Peak calling parameters	Peak calling parameters have been described in the method section.
Data quality	CUTandRUN libraries were assessed stringently by number of peaks and genome browser visualisation.
Software	All software information is described in the method section.

Flow Cytometry

Plots

Confirm that:

☒ The axis labels state the marker and fluorochrome used (e.g. CD4-FITC).

☒ The axis scales are clearly visible. Include numbers along axes only for bottom left plot of group (a 'group' is an analysis of identical markers).

☒ All plots are contour plots with outliers or pseudocolor plots.

☒ A numerical value for number of cells or percentage (with statistics) is provided.

Methodology

Sample preparation	Aggregates were trypsinized with Trypsin/EDTA (0.25%, Thermo Fisher) at 37 degrees for 5-15 min and single cell suspension was incubated with conjugated antibodies for 30 min at room temperature.
Instrument	BD LSRFortessa Cell Analyzer (BD Bioscience)
Software	FlowJo
Cell population abundance	At least 10000 live single cell population was analyzed. The abundance of key populations is reported in each figure panels.
Gating strategy	Cell populations were gated first based on the SSC-A/FSC-A to exclude cell debris and dead cells, followed by gating single cell population based on FSC-A/FSC-W and live cell population based on DAPI

☒ Tick this box to confirm that a figure exemplifying the gating strategy is provided in the Supplementary Information.
