# Supplementary material for: DMRT1 regulates human germline commitment
Source: Nat Cell Biol. 2023 Sep 14;25(10):1439–52. doi: 10.1038/s41556-023-01224-7 (PMC10567552; doi:10.1038/s41556-023-01224-7)

# Source Data for Extended Data Figure 1D: Agarose gel

## DMRT1 locus

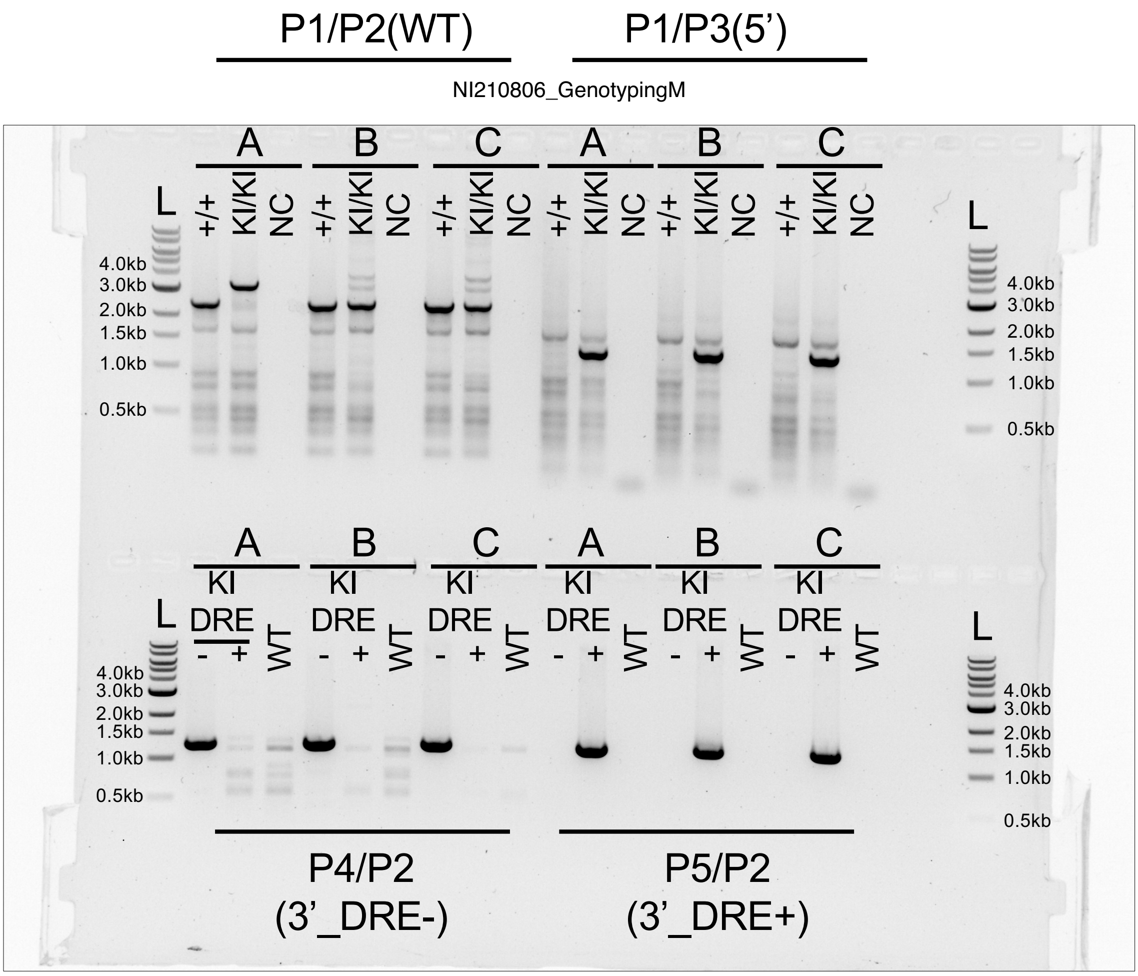


Location: /Users/level2geldoc/Desktop/Naoko
Printed: 06/08/2021 09:25 am

Page 1 of 1

A: WIS2 NANOS3-tdTomato, DMRT1-mVenus
B: WIS2 NANOS3-mVenus, DMRT1-tdTomato
C: Shef-6 DMRT1-tdTomato

L: DNA ladder 1kb

# Source Data for Extended Data Figure 1D: Agarose gel

NANOS3 locus

NI210806_GenotypingN_W_5

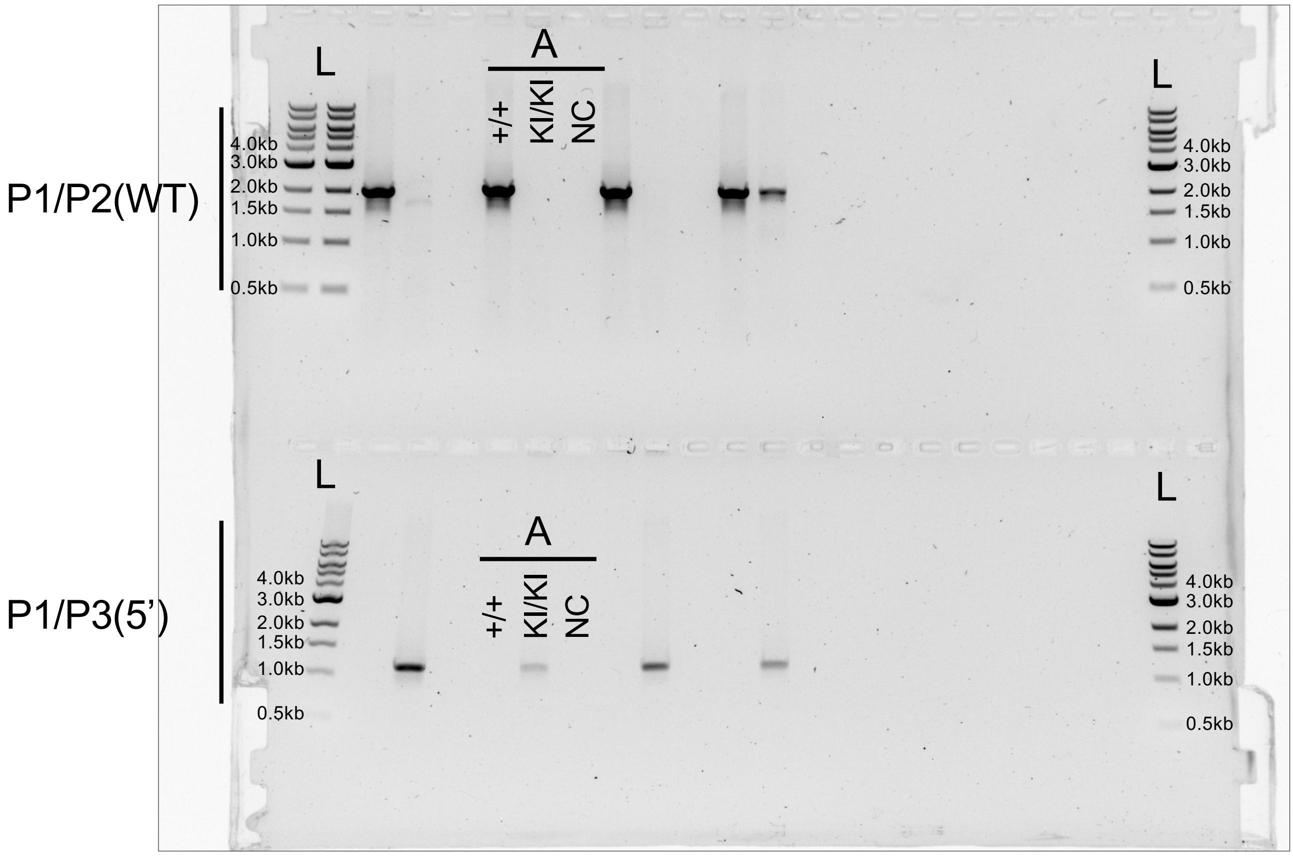


Location: /Users/level2geldoc/Desktop/Naoko
Printed: 06/08/2021 09:32 am



A: WIS2 NANOS3-mVenus, DMRT1-tdTomato

L: DNA ladder 1kb

# Source Data for Extended Data Figure 1D: Agarose gel

NANOS3 locus

NI210808_GenotypingN_3ba

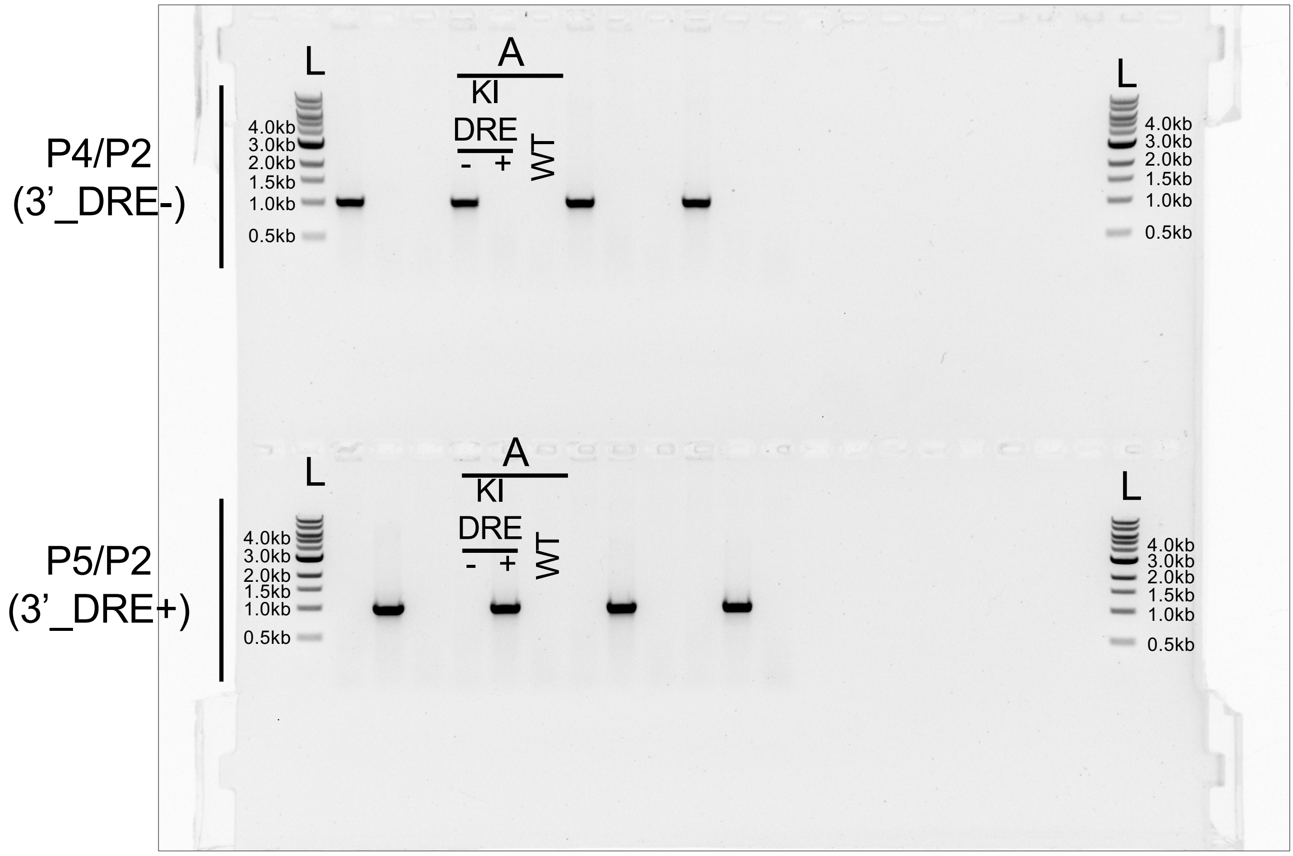


Location: /Users/level2geldoc/Desktop/Naoko
Printed: 08/08/2021 11:03 am

Page 1 of 1

A: WIS2 NANOS3-mVenus, DMRT1-tdTomato

L: DNA ladder 1kb

# Source Data for Extended Data Figure 1D: Agarose gel

## DAZL locus

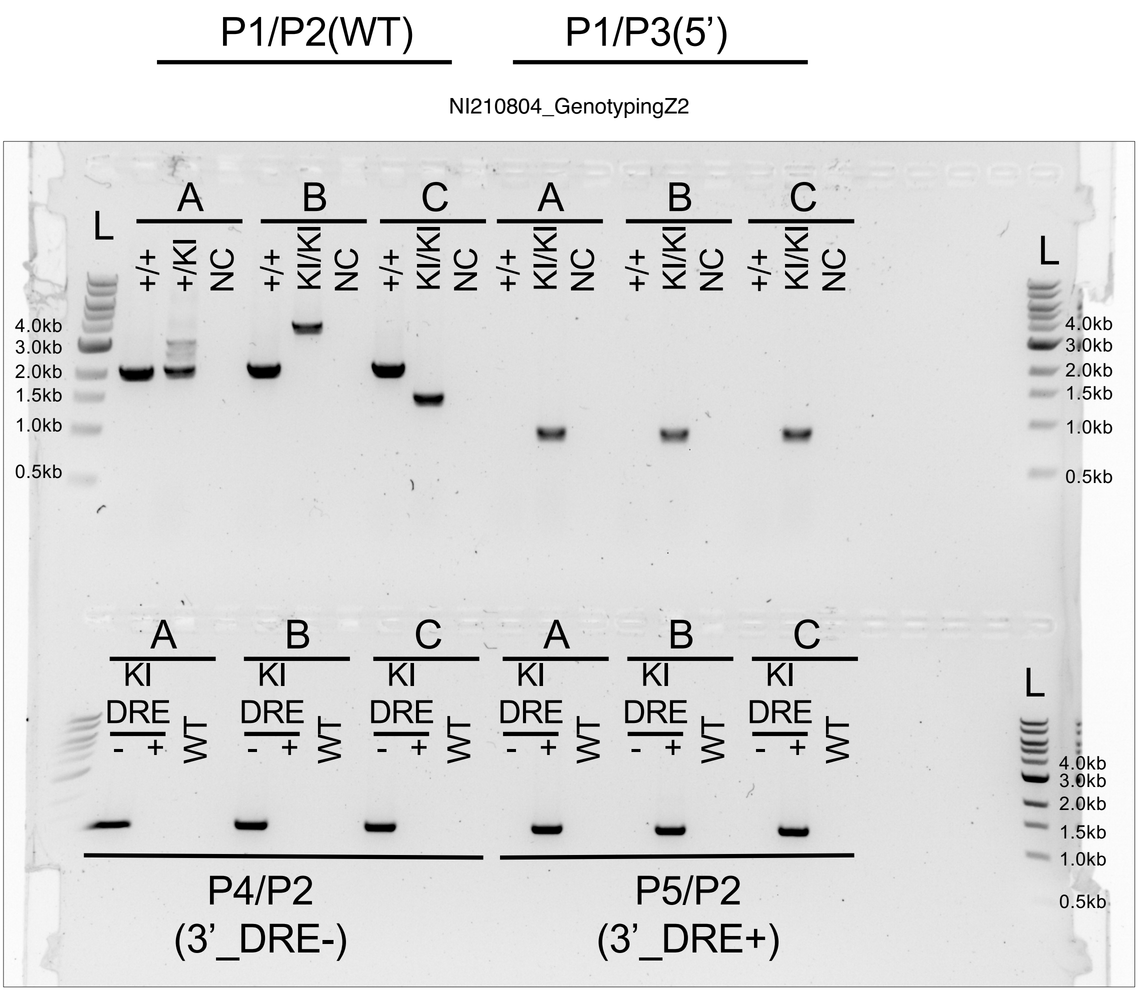


Location: /Users/level2geldoc/Desktop/Naoko
Printed: 04/08/2021 09:41 am

Page 1 of 1

A: WIS2 DAZL-mVenus
B: WIS2 DAZL-tdTomato
C: Shef-6 DAZL-tdTomato

L: DNA ladder 1kb

# Source Data for Extended Data Figure 1G: Western blot

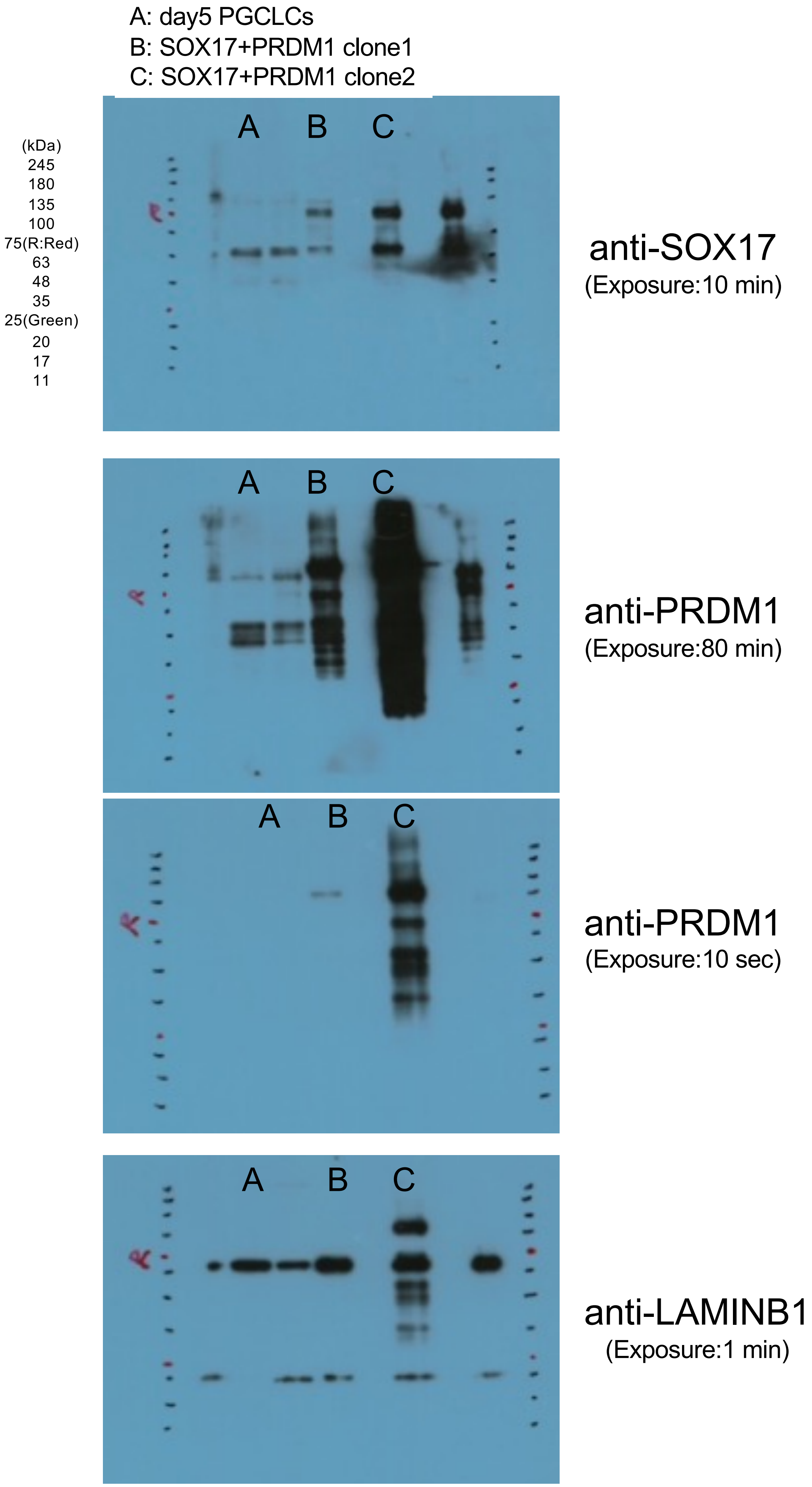

Supplement: Supplementary file 8 — Unprocessed western blots and agarose gel images. [file 41556_2023_1224_MOESM8_ESM.pdf]
