# Supplementary material for: DMRT1 regulates human germline commitment
Source: Nat Cell Biol. 2023 Sep 14;25(10):1439–52. doi: 10.1038/s41556-023-01224-7 (PMC10567552; doi:10.1038/s41556-023-01224-7)
Supplement: Supplementary file 11 — Unprocessed western blots and agarose gel images. [file 41556_2023_1224_MOESM11_ESM.pdf]

Source Data for Extended Data Figure 3E: Western blot

A: ESC
B: day3+6 PGCLC
C: SOX17+DMRT1 clone1

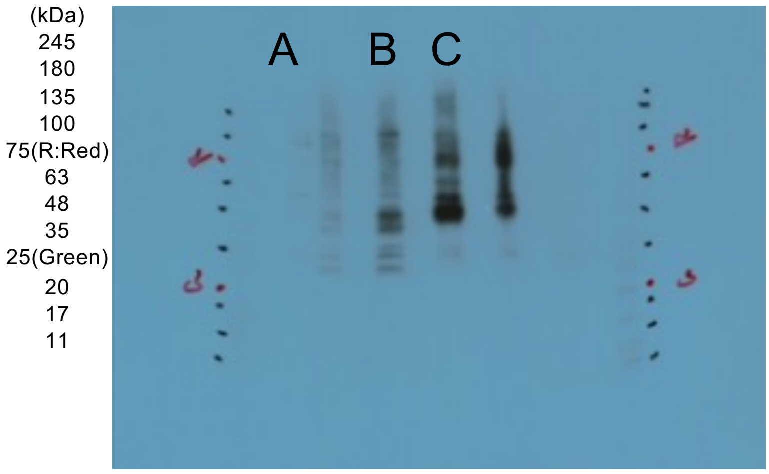

anti-SOX17
(Exposure:10 min)

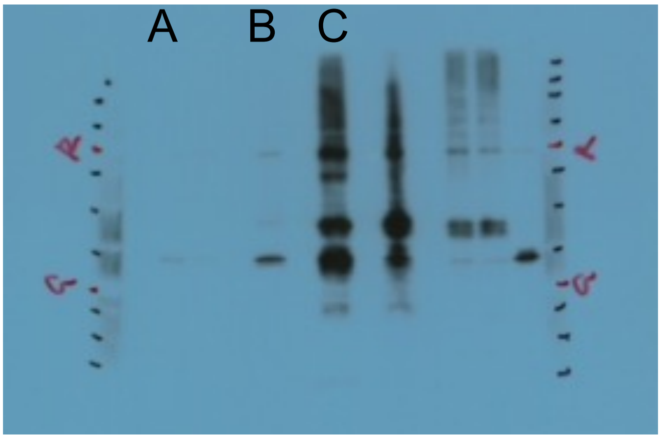

anti-DMRT1
(Exposure:3 sec)

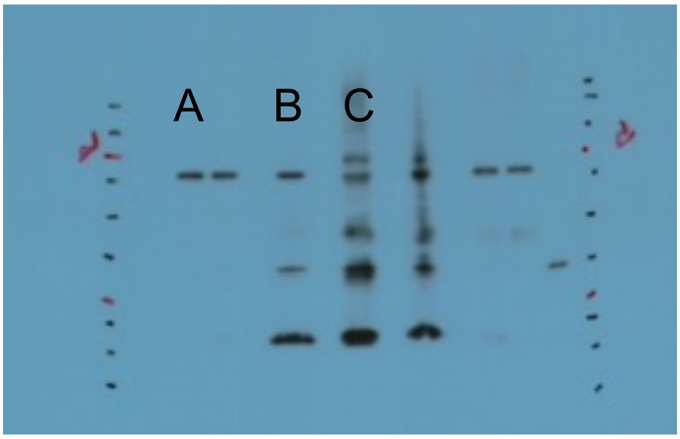

anti-LAMINB1
(Exposure:10 sec)

Source Data for Extended Data Figure 3E: Western blot

A: ESC
B: day3+6 PGCLC
C: SOX17+DMRT1 clone2

(kDa)
245
180
135
100
75(R:Red)
63
48
35
25(Green)
20
17
11

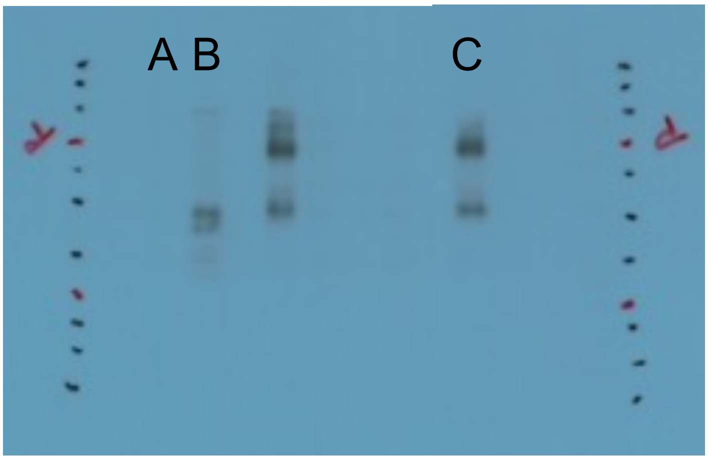

anti-SOX17
(Exposure:15 sec)

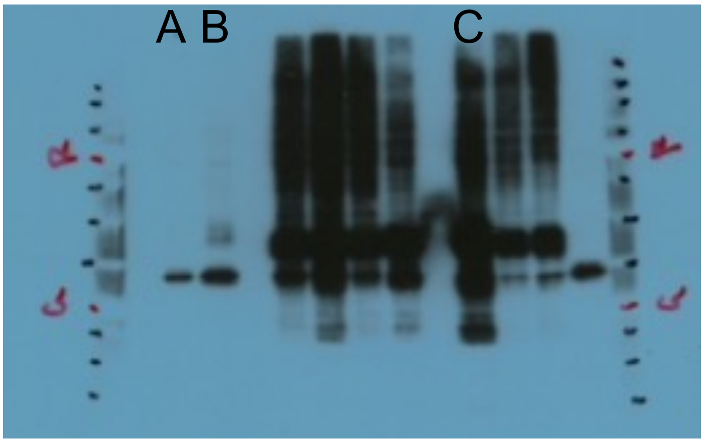

anti-DMRT1
(Exposure:1 mins)

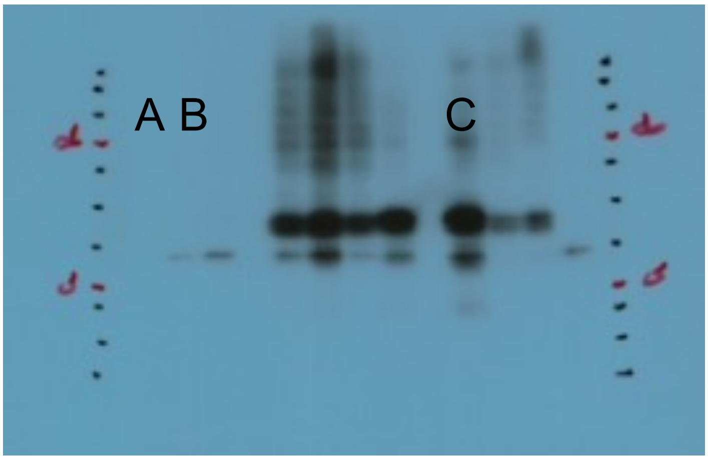

anti-DMRT1
(Exposure:3 sec)

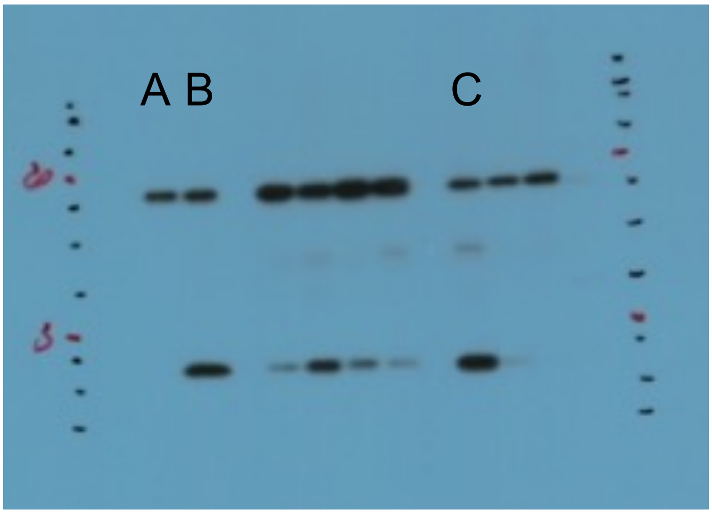

anti-LAMINB1
(Exposure:1 min)
